# Efficacy and Safety of Transcatheter Tricuspid Valve Replacement in Patients With Moderate to Severe Tricuspid Regurgitation: A Systematic Review and Meta‐Analysis on Clinical Outcomes and Echocardiographic Indices

**DOI:** 10.1002/hsr2.70950

**Published:** 2025-06-23

**Authors:** Pouria Azami, Alireza Hosseinpour, Jahangir Kamalpour, Farshad Rajabi, Iman Razeghian‐Jahromi, Sarvenaz Farhangdoost, Reza Golchin Vafa, Ghazaleh Bagheri

**Affiliations:** ^1^ Department of Cardiovascular Medicine, School of Medicine Shiraz University of Medical Sciences; ^2^ School of Medicine Shiraz University of Medical Sciences Shiraz Iran; ^3^ Cardiovascular Research Center, School of Medicine Shiraz University of Medical Sciences Shiraz Iran; ^4^ Clinical Biochemistry Department, School of Medicine Lorestan University of Medical Sciences

**Keywords:** heart valve diseases, transcatheter tricuspid valve replacement, tricuspid regurgitation

## Abstract

**Background and Aims:**

Tricuspid regurgitation (TR) is a prevalent, often overlooked condition linked to significant morbidity and mortality in older adults. Due to the high risks associated with conventional surgery, transcatheter tricuspid valve replacement (TTVR) has emerged as a less invasive alternative. This systematic review and meta‐analysis evaluated the clinical outcomes and echocardiographic indices of TTVR.

**Methods:**

Five databases were searched systematically, and eligible studies included patients with moderate or severe TR who underwent TTVR. Risk of bias was assessed using the ROBINS‐I tool for observational studies and the JBI checklist for case series. A random‐effects meta‐analysis was performed to evaluate the impact of TTVR on major adverse cardiovascular events (MACE) and echocardiographic parameters.

**Results:**

Twenty‐one studies with 643 patients (mean age 75.8 years, 70.76% female) reported a 94% technical success rate for TTVR (95% CI: 91–96%). 30‐day mortality was 4% (95% CI: 2–6%) and 1‐year mortality was 9% (95% CI: 6%–13%). Significant improvements were noted in TR severity (OR = 0.0013, 95% CI: 0.0006–0.0027, *p* < 0.001) and PASP (MD = −8.69 mmHg, 95% CI: −11.54 to −5.84, *p* < 0.001), along with reductions in right ventricular base diameter (MD = −6.33 mm, 95% CI: −8.92 to −3.75, *p* < 0.001) and RV end‐diastolic mid diameter (MD = −6.33 mm; 95% CI [−8.18, −5.52]; *p* < 0.001; I² = 5%).

**Conclusion:**

TTVR presents a promising treatment alternative for high‐risk patients with severe TR, demonstrating high technical success, favorable clinical outcomes, and significant echocardiographic improvements. While the procedure is associated with low in‐hospital and 1‐year mortality, further studies are needed to evaluate long‐term outcomes and optimize patient selection for this emerging therapy.

**Abbreviations**
AF: atrial fibrillation
AKI: acute kidney injury
CAD: coronary artery disease
CI: confidence interval
CKD: chronic kidney disease
COPD: chronic obstructive pulmonary disease
EROA: effective regurgitant orifice area
FAC: fractional area change
HF: heart failure
KCCQ: Kansas City Cardiomyopathy Questionnaire
LVEF: left ventricular ejection fraction
MACE: major adverse cardiovascular events
MD: mean difference
MI: myocardial infarction
MWT: 6‐min walk test
NYHA: New York Heart Association
OR: odds ratio
PASP: pulmonary artery systolic pressure
RA: right atrium
RV: right ventricle
SD: standard deviation
STS: Society of Thoracic Surgeons
TAPS: tricuspid annular plane systolic excursion
TEER: transcatheter edge‐to‐edge repair
TIA: transient ischemic attack
TR: tricuspid regurgitation
TTVR: transcatheter tricuspid valve replacement
VARC: valve academic research consortium

## 1 Introduction

Tricuspid regurgitation (TR) is a valvular heart condition that often coexists with other valvular diseases, leading to its designation as the ‘forgotten valve’ due to historically being overlooked in clinical practice [1, 2]. Trivial TR was observed in 70% of the normal population [3], while clinically significant TR (i.e., moderate or severe) was present in up to 5.6% of women and 1.5% of men older than 70 years, according to the Framingham Heart Study [4], which is linked to unfavorable clinical outcomes and elevated mortality rates [5]. The etiology of TR is divided into the less common primary TR (8–10% of all cases), caused by intrinsic valve disease, and the more common secondary TR, which results from issues with the right atrium, tricuspid annulus, or right ventricle that lead to leaflet malcoaptation [1, 6].

The therapy should be initiated before the onset of tricuspid valve tethering, increased left atrial volume, enlarged tricuspid annular diameter, and right ventricular remodeling, as these factors are predictors of severe TR [1, 5]. According to current guidelines, medical therapy for TR is limited to diuretics and treating the underlying causes of secondary TR [7], primarily to manage congestive symptoms [8]. However, mechanical correction is the only proven effective treatment for secondary TR apart from addressing any underlying conditions [9]. Conventionally, TR has been managed through surgical intervention to repair or replace the damaged valve. Despite its prevalence and the significant number of patients with moderate to severe TR, only a limited number of these surgeries are performed annually, possibly due to the high surgical risk for many patients [10].

Transcatheter‐based techniques have emerged as an alternative treatment option for patients ineligible for traditional surgical interventions, including leaflet‐directed interventions, annular reshaping, and transcatheter valve replacement [11]. Although valve repair with an annuloplasty ring is the preferred procedure, valve replacement becomes necessary in cases with significant leaflet tethering, right ventricular dysfunction, and a large annular diameter, which are characteristics of secondary TR [7, 12, 13]. Additionally, replacement may be more appropriate when repair is unlikely to fully resolve TR due to factors like a large coaptation gap, noncentral regurgitant jets, or calcification [14]. Early feasibility studies have demonstrated that orthotopic tricuspid valve replacement is a feasible procedure with significant efficacy in reducing tricuspid regurgitation. Clinical outcomes suggest it can also improve survival rates compared to traditional repair methods [15].

TTVR devices are classified into orthotopic (e.g., NaviGate, EVOQUE, Trisol, LUX‐Valve, Intrepid, TRICares), positioned at the tricuspid valve annulus, and heterotopic (e.g., Sapien, TricValve, TriCento), implanted in the vena cava to mitigate the hemodynamic effects of tricuspid regurgitation [14]. Orthotopic devices are preferred for direct intervention at the tricuspid valve annulus when anatomically possible, while heterotopic devices are used when such placement is impractical due to anatomical constraints [14]. TTVR, inspired by advancements in aortic and mitral valve treatments [16], represents a novel approach to tricuspid regurgitation, offering distinct advantages over traditional open surgery and repair techniques. Despite its potential benefits, the long‐term outcomes of TTVR have not yet been extensively evaluated. This study aims to pool results from various studies to assess the clinical outcomes, echocardiographic measurements, and other relevant aspects of this emerging procedure.

## 2 Methods

### 2.1 Data Source and Search Strategy

This systematic review and meta‐analysis has been registered on Prospero (ID: CRD42024561137) [17]. It was conducted following the established practices recommended by the Preferred Reporting Items for Systematic Reviews and Meta‐Analyses (PRISMA) guidelines [18]. We systematically searched electronic databases including PubMed, Scopus, Web of Science, Embase, and Google Scholar for studies published on or before March 15, 2024. The search results were updated on April 26, 2025, to ensure the most recent evidence was captured. We used “AND” and “OR” as Boolean operators in our search strategy. Standardized methods were utilized for searching the following keywords: “tricuspid,” “tricuspid valve,” “tricuspid valve replacement,” “tricuspid valve intervention,” “tricuspid valve implantation,” “transcatheter tricuspid replacement,” “transcatheter tricuspid intervention,” “transcatheter tricuspid implantation,” “transcatheter tricuspid valve replacement,” “transcatheter tricuspid valve implantation,” “transcatheter tricuspid valve intervention,” “TTVR,” and “TTVI,”. Additionally, two authors manually searched the references of reviews, case series, case reports, and editorials, and included studies to find relevant studies. Records from major scientific conferences were reviewed to uncover gray literature. The search included all languages and time periods. The comprehensive search strategy is mentioned in Supplementary Table 1.

### 2.2 Study Selection and Inclusion Criteria

EndNote reference management software (version 20.2.1, Clarivate Analytics) was utilized to identify and remove duplicate entries. Two independent authors (PA and FR) reviewed the final list of articles. A third investigator (AH) mediated and resolved any disagreements in article selection. Studies were considered eligible if they met all of the following criteria [1]: the study population consisted of patients with moderate or severe TR who had undergone TTVR [2], the studies included four or more patients [3], patients were aged 18 years or older, and [4] the studies reported at least one of the primary outcomes.

The primary outcomes included [1] the incidence of major adverse cardiovascular events (MACE), defined as composite events of myocardial infarction (MI), stroke, and cardiac mortality, and [2] changes in echocardiographic measures, including tricuspid regurgitation (TR) severity, tricuspid annular plane systolic excursion (TAPS), pulmonary artery systolic pressure (PASP), right ventricular (RV) diameter, and RV fractional area change (FAC). Secondary outcomes included [1], technical success as a key procedural outcome, while procedural aspects such as procedure duration, hospital stays, and access route were noted as influencing factors [2]; quality of life measures including New York Heart Association (NYHA) functional class, the 6‐min walk test (6MWT), and the Kansas City Cardiomyopathy Questionnaire (KCCQ) [3]; procedural complications like bleeding, access site issues, vascular complications requiring reintervention, conversion to surgery, and acute kidney injury (AKI) [4]; long‐term adverse events such as device‐related pulmonary embolism, device migration, hospitalization due to heart failure (HF), and pulmonary complications; and [5] additional changes in echocardiographic parameters, including left ventricular ejection fraction (LVEF), right atrial (RA) volume, and tricuspid valve (TV) mean gradient. Success was defined as effective device deployment with no clinically significant paravalvular leak detected by transthoracic echocardiography (TTE) at discharge [19]. We obtained the longest follow‐up duration available for each outcome from each study.

The exclusion criteria included studies with patients who underwent valve‐in‐ring, valve‐in‐valve, and heterotopic transcatheter tricuspid valve replacement (TTVR), as well as those with previous tricuspid valve interventions such as failed surgical annuloplasty rings or structural dysfunction of bioprostheses. Studies involving patients who underwent surgical intervention instead of transcatheter procedures, or repair instead of replacement, were also excluded. Additionally, studies with incomplete baseline or follow‐up outcomes were excluded, as accurate analysis was impossible.

### 2.3 Data Extraction and Quality Appraisal

Two authors (PA and FR) reviewed and extracted pre‐specified data from the included articles. The following information was collected: study characteristics such as first author's name, publication year, country (or countries) where the studies were conducted, study design, and follow‐up time; study population characteristics such as age, gender, sample size, baseline comorbidities (including diabetes mellitus (DM), chronic kidney disease (CKD), chronic obstructive pulmonary disease (COPD), coronary artery disease (CAD), atrial fibrillation (AF), ascites, and edema), and surgery risk scores (STS score and EuroSCORE II); as well as primary and secondary outcomes of interest All included studies received the required ethical approval.

To assess the quality of the observational studies, we used the ROBINS‐I tool (Risk Of Bias In Non‐randomized Studies of Interventions), which evaluates the risk of bias across seven domains: bias due to confounding, bias in participant selection, bias in classification of interventions, bias due to deviations from intended interventions, bias due to missing data, bias in measurement of outcomes, and bias in selection of the reported result. Each study was rated for the risk of bias as low, moderate, high, or critical [20]. For case series studies, we employed the JBI critical appraisal tool to assess the risk of bias [21].

### 2.4 Statistical Analysis

All the statistical analyses done in this study were performed using R Software Version 4.3.2. For estimating the proportion of patients experiencing clinical outcomes such as mortality and other adverse events, the event rate and the total sample size were used to pool the overall incidence rate with a 95% confidence interval (CI). For other endpoints, a comparison was made between before and after performing the intervention. The odds ratio (OR) was set as the effect size for binary endpoints, and the mean difference (MD) was reported comparing the post‐ and before‐intervention data for continuous outcomes. The random effects model was used for all the analyses to address the potential sources of heterogeneity among the studies. I² and the associated *p*‐value were used to assess the amount of heterogeneity, and in case of a *p*‐value < 0.1, significant heterogeneity was noted. To evaluate the publication bias, Egger's test and visualization of the funnel plot were used for analyses including 10 or more studies. In case of a significant Egger's test or asymmetry in the funnel plot, the trim‐and‐fill method was used to regenerate and report the results after adjusting the analyses to fix the publication bias. The mean and standard deviation (SD) were used in this study, and the formula by Wan et al. was applied to convert median and interquartile range values to the mean and SD [22]. The analysis results were considered statistically significant if the effect size and the associated CI did not cross the null zone (*p* < 0.05).

## 3 Results

**Figure 1:**
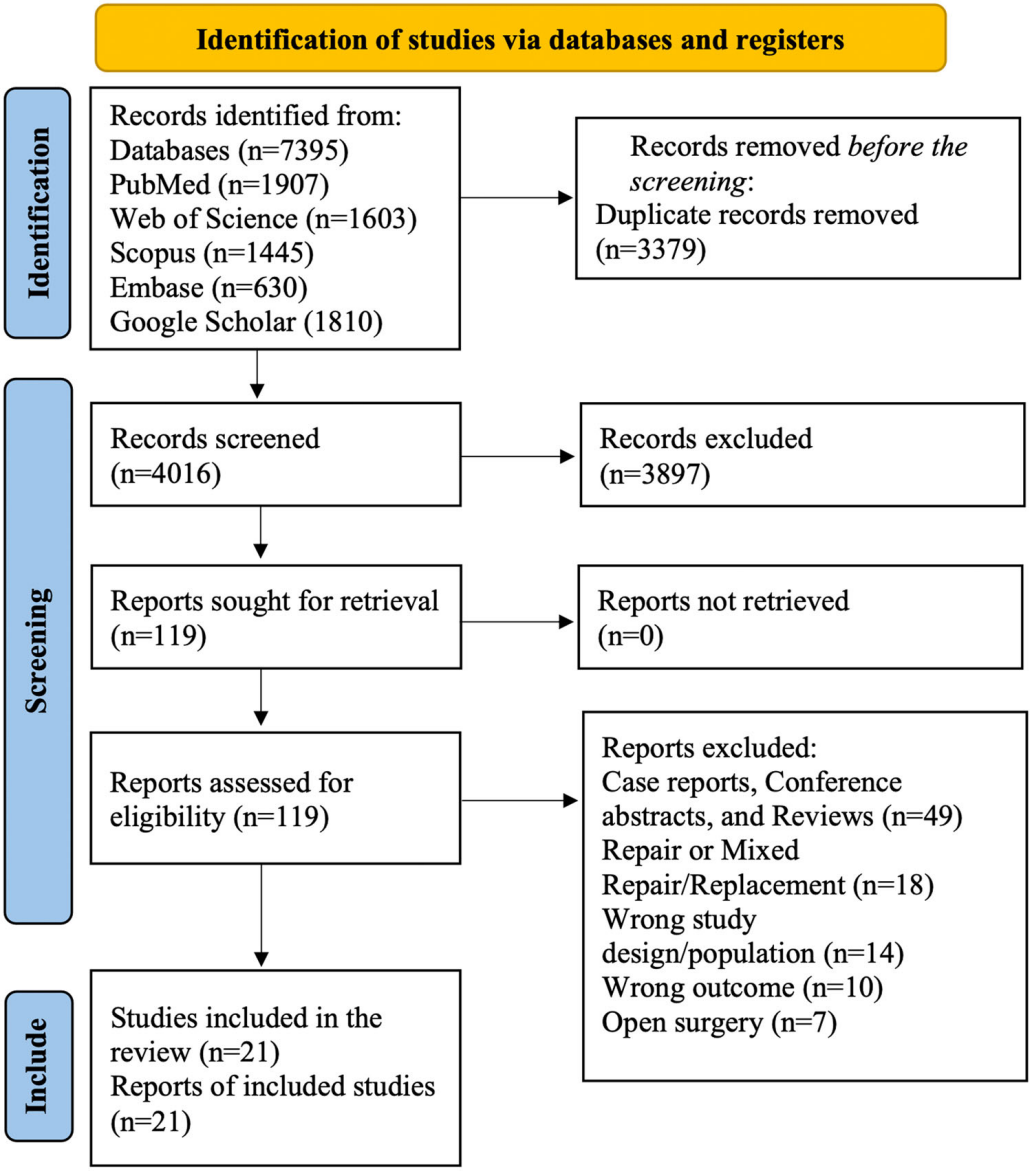
PRISMA flowchart depicting the literature search, screening, and selection process.

An initial search across five electronic databases initially identified 7395 studies, which were reduced to 4016 after removing duplicates. From these, 3897 studies were excluded based on their titles and abstracts. The remaining 119 studies underwent full‐text screening, leading to the selection of 21 articles, which included 13 observational studies, 4 case series studies, and 4 editorial letters for this meta‐analysis, as depicted in Figure 1. The search strategy and PRISMA checklist for this study are available in Supplementary Tables 1 and 2 [18].

### 3.1 Baseline Characteristics

This present meta‐analysis involved 21 studies with 643 patients [15, 23, 24, 25, 26, 27, 28, 29, 30, 31, 32, 33, 34, 35, 36, 37, 38, 39, 40, 41, 42]. Patients had a mean age of 75.8 years (95% CI: 69.4−75.8 years), including 70.76% (68.17–73.23%) females. They were high surgical risk, with a mean EuroSCORE II score of 8.0% (95% CI: 6.9–9.1%) and a mean STS score of 9.0% (95% CI: 8.0–10.0%). Severe or grade ≥ 3TR was diagnosed in 99.5% of patients (95% CI: 97.8–100.0%), and pathological TR (secondary TR) was diagnosed in 75.33% (95% CI: 67.69–81.65). 89.83 (95% CI: 83.69–93.83) of patients were in NYHA functional class III or IV at the baseline. The mean pooled TAPSE (mm) was 14.78 mm (95% CI: 14.06–15.51), RV FAC% was 38.44% (95% CI: 36.35–40.52), TR EROA was 86.94 (95% CI: 74.58–99.31) and LVEF% was 56.19% (95% CI: 53.52–58.86) at the baseline. All baseline characteristics, including detailed echocardiographic parameters, are provided in Tables 1 and 2, as well as Supplementary Table 3.

**Table 1:** Baseline characteristics.

First Author, year	Sample size (No)	Age (years)	Female (No)	DM (No)	HTN (No)	HLP (No)	COPD (No)	CKD (No)	AF (No)	CAD (No)	Edema (No)	Ascites (No)	Pulmonary hypertension (No)	Functional TR (secondary TR) (No)	NYHA functional class III or IV (No)	6MWD (m)	EUR score II (%)	Kansas City questionnaire score	Prior valvular surgery (No)	Prior MI (No)	Prior CVA/TIA	Prior CABG (No)	Prior PCI (No)	Prior PPM/ICD (No)
Kodali, 2023 [40]	176	78.7 ± 7.33	125	36	148	115		103	162	36	65	39	132	120	131	214.3 ± 108	5.1 ± 4	46 ± 21.8	79	14	24	29		57
Kodali, 2022 [41]	56	79.3 ± 7.7	43	12	49	39	10	37		8	20	12	45	38	49	199.1 ± 128.6	5.6 ± 4.9	46.5 ± 23.1	22	2	15	9	8	19
Dershowitz, 2023 [42]	25	77.5 ± 11.7	16																					
Fam, 2021 [43]	25	76 ± 3	22	8	17		3	15	21	7	18	14		19	24		7.7 ± 2.2		52	1	6	5	2	9
Hagemeyer, 2024 [44]	38	79.83 ± 5.5	26		26		2	26	36		1	6		32	30				9			8	9	10
Hahn, 2020 [45]	30	76 ± 7.78	17	11	21	17		19	27				9		26		10.79 ± 5.41		12	8	2	10	5	9
Lu, 2021 [46]	46	67.26 ± 10.86	34	13			18	28	41	8	46	22	24		46	211.6 ± 70.25	10.3 ± 3.5	33 ± 7.65	28		5	3		12
Mao, 2022 [47]	15	65.33 ± 13.08	9	5		9	6	7	13	2	15	7	8		15	211.66 ± 64.05	9.5 ± 3.43	32.33 ± 10.63	11		1	2		6
Ning, 2023 [48]	22	64.8 ± 8.2	14	2	7		1	1	18	4			15		22	282.3 ± 76.1		49.1 ± 13.6	18	0	3	1	1	4
Wang, 2024 [49]	20	65.7 ± 7.4	13				8		17	6	20	12	11		20	209.5 ± 56.3		35.9 ± 6.7	9	4	3	4 (CABG or PCI)	4 (PCI or CABG)	7
Webb, 2022 [15]	27	77 ± 8	24	8	19		3	15	23	8	19	14		19	24	285 ± 105	7.4 ± 5.1		11		6	5	3	9
Wei, 2022 [50]	30	65.2 ± 7.9	19	3	6				25	4	23				30	259.3 ± 70.3		49 ± 13	24		3	1	1	5
Yu, 2022 [51]	17	68 ± 9.34	15	5	4	3	8	4	14	1			11		17	352.53 ± 46.07	7.33 ± 2.42	59.66 ± 26.59			1			
Hahn, 2019 [52]	5	84.4 ± 6.28	2	3	4			3	5	1					4				1		2	3		
Elgharably 2019 [53]	4	74.5 ± 7.04	3	1	2		2	2		3												3		1
Mao, 2023 [54]	6	63.16 ± 4.66	4	3			1	6	0	2			3	6	6	201.66 ± 23.73	9.71 ± 1.44	30.5 ± 2.86	4		1	1		
Sun, 2021 [55]	6	56.5 ± 2.36	6						4					6	6		7.78 ± 1.01							
Fam, 2024 [56]	20	79 ± 6	10											18	12									
Lu, 2020 [57]	12	69.66 ± 7.62	7											12	12	251.5 ± 142.56								
Stoltz, 2023 [58]	38	77 ± 12	28								27	19			35		7.9 ± 6.5							
Weckbach, 2023 [59]	25	81 ± 5.87	22												25									

Abbreviations: 6MWT, six‐minute6‐min walk test; AF, atrial fibrillation; CABG, coronary artery bypass graft; CAD, coronary artery disease; CKD, chronic kidney disease; COPD, chronic obstructive pulmonary disease; CVA, cerebrovascular accident; DM, diabetes mellitus; EUR score II, European System for Cardiac Operative Risk Evaluation II; HLP, hyperlipidemia; HTN, hypertension; ICD, implantable cardioverter‐defibrillator; MI, myocardial infarction; No, number; NYHA, New York Heart Association; PPI, prior permanent pacemaker; TIA, transient ischemic attack; TR, tricuspid regurgitation.

**Table 2:** Study and population characteristics.

First Author, year	No. of participants	Study design	Device	Access route	Procedure duration (min)	Technical success (No)	Hospital stays (days)	F/u duration (months)	Outcomes assessed
Kodali, 2023	176	Observational (clinical trial)	EVOQUE	Right femoral vein	121/4 ± 65/7	164	3 ± 1/49	12	mortality: 30 days, 6months, 1year, all cause, cardiac complications: bleeding, device related pulmonary embolism, nonelective TV reintervention, major access site or vascular complication, AKI, TIA/stroke, MI clinical: NYHA functional class, 6MWD, KCCQ, edema, 1year HF hospitalization echocardiographic: TR grade, TAPSE, PASP, RV end‐diastolic mid diameter, RV FAC, LVEF, RA volume, TV mean gradient, IVC diameter
Kodali, 2022	56	Observational	EVOQUE	Right femoral vein		54	4/3 ± 4/3	1	mortality: 30 days, all cause, cardiac complications: bleeding, device related pulmonary embolism, nonelective TV reintervention, major access site or vascular complication, AKI, TIA/stroke, MI, arrhythmia, device migration clinical: NYHA functional class, 6MWD, KCCQ, edema echocardiographic: TR grade, TAPSE, PASP, RV end‐diastolic mid diameter, RV FAC, LVEF, RA volume, TV mean gradient, IVC diameter, TR EROA
Dershowitz, 2023	25	Observational						12	echocardiographic: TAPSE, PASP, RV end‐diastolic mid diameter, RV FAC
Fam, 2021	25	Observational	EVOQUE	Right femoral vein	140 ± 79	23		1	mortality: in‐hospital, 30 days, all cause, cardiac complications: bleeding, nonelective TV reintervention, major access site or vascular complication, AKI, TIA/stroke, MI, arrhythmia clinical: NYHA functional class, edema, ascites, 1year HF hospitalization echocardiographic: TR grade, TAPSE, RV end‐diastolic base diameter, RV FAC, TV mean gradient, IVC diameter
Hagemeyer, 2024	38	Observational	EVOQUE, TOPAZ, CARDIOV ALVE, VDYNE			35	9 ± 6/93	1	mortality: in‐hospital, 30 days, all cause, cardiac complications: bleeding, nonelective TV reintervention, major access site or vascular complication, AKI, TIA/stroke, MI clinical: NYHA functional class, 1year HF hospitalization echocardiographic: TR grade, TAPSE, RV end‐diastolic mid diameter, RV end‐diastolic base diameter, RV FAC, LVEF, IVC diameter, TR EROA
Hahn, 2020	30	Observational	GATE	Transatrial (25), Transjugular (5)	157/66 ± 82/51	26	14/16 ± 11/67	1	mortality: in‐hospital, 30 days, all cause, cardiac complications: bleeding, device related pulmonary embolism, nonelective TV reintervention, device migration, AKI, TIA/stroke, MI clinical: NYHA functional class Echocardiographic: TR grade, TAPSE, PASP, LVEF, PISA radius, TV mean gradient, TR EROA
Lu, 2021	46	Observational	LuX‐Valve	Transatrial (minimally invasive thoracotomy)	149/6 ± 46/8	45	13/66 ± 8/41	6	mortality: in‐hospital, 6months, all cause complications: bleeding, device related pulmonary embolism, nonelective TV reintervention, major access site or vascular complication, AKI, TIA/stroke, MI, valve thrombosis, device migration clinical: NYHA functional class, 6MWD, KCCQ, edema, 1year HF hospitalization echocardiographic: TR grade, TAPSE, RV end‐diastolic base diameter, RV FAC, LVEF, RV volume, RA volume, TV mean gradient, TR EROA
Mao, 2022	15	Observational	LuX‐Valve	Transatrial (minimally invasive thoracotomy)	136/66 ± 38/22	15	13 ± 9/5	12	mortality:30 days, 6months, 1year, all cause, cardiac complications: bleeding, device related pulmonary embolism, nonelective TV reintervention, major access site or vascular complication, AKI, TIA/stroke, MI, valve thrombosis, arrhythmia, infection, pulmonary complication clinical: NYHA functional class, 6MWD, KCCQ, edema, 1year HF hospitalization, ascites echocardiographic: TR grade, TAPSE, RV end‐diastolic mid diameter, RV end‐diastolic base diameter, RV FAC, RA volume, RV volume, TV mean gradient, TR EROA
Ning, 2023	22	Observational	LuX‐Valve	Transatrial (minimally invasive thoracotomy)		22	13/26 ± 6/89	6	complications: bleeding, device related pulmonary embolism, nonelective TV reintervention, major access site or vascular complication, AKI, TIA/stroke, MI, arrhythmia, post‐op pulmonary complication, infection clinical: NYHA functional class echocardiographic: TR grade, TAPSE, RV FAC, LVEF, tricuspid annulus diameter, RA volume, IVC diameter, TR EROA,
Wang, 2024	20	Observational	LuX‐Valve (plus)	Transatrial (11) and transjugular (9)	133 ± 28/1	20	9/8 ± 3/3	6	mortality: in‐hospital, 30 days, 6months, all cause, cardiac complications: bleeding, device related pulmonary embolism, nonelective TV reintervention, major access site or vascular complication, AKI, TIA/stroke, MI, pulmonary complication, infection, valve thrombosis, arrhythmia clinical: NYHA functional class, 6MWD, KCCQ, 1year HF hospitalization echocardiographic: TR grade, TAPSE, PASP, RV end‐diastolic mid diameter, RV end‐diastolic base diameter, RV FAC, LVEF, RA volume, IVC diameter
Webb, 2022	27	Observational	EVOQUE	Right femoral vein		27		12	mortality:30 days, 6months, 1year, all cause, cardiac complications: bleeding, device related pulmonary embolism, nonelective TV reintervention, major access site or vascular complication, AKI, TIA/stroke, MI, arrhythmia, infection clinical: NYHA functional class, 6MWD, edema, ascites 1year HF hospitalization echocardiographic: TR grade, TAPSE, PASP, RV end‐diastolic mid diameter, RV end‐diastolic base diameter, RV FAC, LVEF, TV mean gradient, IVC diameter
Wei, 2022	30	Observational	LuX‐Valve	Transatrial (minimally invasive thoracotomy)	166/66 ± 31/13	30	24/5 ± 7/8	6	mortality:30 days, 6months, all cause, cardiac complications: bleeding, device thrombosis, nonelective TV reintervention, major access site or vascular complication, pulmonary complication, infection, AKI, TIA/stroke, MI echocardiographic: TAPSE, RV FAC, LVEF, tricuspid annulus diameter, RV volume, RA volume, TV mean gradient, IVC diameter
Yu, 2022	17	Observational	LuX‐Valve	Transatrial (minimally invasive thoracotomy)		17		1	mortality: in‐hospital, 30 days, all cause, cardiac complications: bleeding, valve thrombosis, nonelective TV reintervention, major access site or vascular complication, AKI, TIA/stroke, MI, infection, arrhythmia, pulmonary complication clinical: NYHA functional class, 6MWD, KCCQ echocardiographic: TR grade
Hahn, 2019	5	Case report	GATE	Transatrial (minimally invasive thoracotomy)		5	17/25 ± 15/1	1	mortality: in‐hospital, 30 days, 6months, all cause, cardiac complications: bleeding, AKI, TIA/stroke, MI, valve thrombosis, arrhythmia echocardiographic: TAPSE, RV end‐diastolic mid diameter, RV end‐diastolic base diameter
Elgharably 2019	4	Case report	GATE	Transatrial (minimally invasive thoracotomy)		4	12/5 ± 11	9	mortality: in‐hospital, 30 days, 6months, all cause, cardiac complications: bleeding, valve thrombosis echocardiographic: TR grade
Mao, 2023	6	Case report	LuX‐Valve	Transatrial (minimally invasive thoracotomy)	134/16 ± 10/68	6	11/66 ± 2/98	24	mortality: in‐hospital, 30 days, 6months, 1year, all cause, cardiac complications: bleeding, device related pulmonary embolism, nonelective TV reintervention, major access site or vascular complication, AKI, TIA/stroke, MI, device migration, arrhythmia, infection clinical: NYHA functional class, 6MWD, KCCQ, 1year HF hospitalization echocardiographic: TR grade, TAPSE, RV end‐diastolic mid diameter, RV end‐diastolic base diameter, RV FAC, LVEF, RV volume, RA volume, TR EROA, TV mean gradient
Sun, 2021	6	Case report	LuX‐Valve	Transatrial (minimally invasive thoracotomy)		6	8/66 ± 3/29	12	mortality:30 days, 6months, 1year, all cause, cardiac complications: bleeding, device related pulmonary embolism, nonelective TV reintervention, major access site or vascular complication, AKI, TIA/stroke, MI, device thrombosis, arrhythmia, infection, pulmonary complication clinical: NYHA functional class, 1year HF hospitalization echocardiographic: TR grade, TAPSE, LVEF, TR EROA TV mean gradient
Fam, 2024	20	Editorial letter	Cardiovalve			18		1	mortality: in‐hospital, 30 days, all cause, cardiac complications: bleeding, device related pulmonary embolism, nonelective TV reintervention, major access site or vascular complication, AKI, TIA/stroke, MI, device thrombosis, infection, pulmonary complication clinical: NYHA functional class, HF hospitalization echocardiographic: TR grade, RV end‐diastolic base diameter
Lu, 2020	12	Editorial letter	LuX‐Valve	Transatrial (minimally invasive thoracotomy)		12		1	mortality:30 days, all cause, cardiac complications: bleeding, device related pulmonary embolism, nonelective TV reintervention, major access site or vascular complication, AKI, TIA/stroke, MI, arrhythmia, device migration clinical: NYHA functional class, 6MWD, 1year HF hospitalization echocardiographic: TR grade, TAPSE, PASP, RV end‐diastolic mid diameter, RV FAC, LVEF, tricuspid annulus diameter, PISA radius, RA volume, TV mean gradient, IVC diameter
Stoltz, 2023	38	Editorial letter	EVOQUE			38		24	mortality: in‐hospital, 1year, 2years, all cause, cardiac complications: bleeding, device related pulmonary embolism, nonelective TV reintervention, major access site or vascular complication, AKI, TIA/stroke, MI, valve thrombosis, device migration, arrhythmia clinical: NYHA functional class, edema, ascites
Weckbach, 2023	25	Editorial letter	EVOQUE			25			complications: valve thrombosis clinical: NYHA functional class Echocardiographic: TR grade

*Note:* No: number; f/u: follow‐up; min: minutes.

### 3.2 Procedural Features

The TTVR procedure was performed successfully in 94% of the cases (events/total = 592/618; 95% CI [0.91, 0.96]; I² = 0%) [15, 23, 24, 25, 26, 27, 28, 29, 30, 31, 32, 33, 34, 35, 36, 37, 38, 39, 40, 42]. The pooled mean duration of the procedure was 141.51 min (95% CI [130.21, 152.80]; I² = 83%) [23, 24, 26, 27, 28, 29, 38, 39], and the hospital stay was 11.5 days (95% CI [8.31, 14.68]; I² = 98%) [24, 25, 26, 27, 28, 29, 31, 32, 34, 37, 38, 39, 40] (Supplementary Figure 1). Various devices were utilized during the procedures, with the most commonly used being the EVOQUE in 54% of cases, followed by the LuX‐Valve in 27% of cases. The access routes comprised transatrial, transfemoral, and transjugular approaches. The follow‐up period for the included studies ranged from 1 month to 24 months, with a median follow‐up duration of 6 months. Table 3 shows the details of the included studies and procedural features.

**Table 3:** Baseline echocardiographic characteristics.

First Author, year	TR severe or greater than grade II or III (No)	RV volume (mL)	RA volume (mL)	TAPSE (mm)	PASP (mm)	RV end‐diastolic base diameter (mm)	RV end‐diastolic mid diameter (mm)	RV FAC (%)	LVEF (%)	Tricuspid annulus diameter (mm)	PISA radius (mm)	TR EROA (mm2)	TV mean gradient (mm hg)	IVC diameter (mm)
Kodali, 2023	155		144.4 ± 54.1	16 ± 3	39.3 ± 12.8		41.4 ± 8.8	38.7 ± 10.1	54.1 ± 11.2				1.7 ± 1	27.6 ± 7.7
Kodali, 2022	51		154.1 ± 66.1	13.7 ± 1.8	40.1 ± 10.5		33.1 ± 7.2	37.1 ± 9.2	53.4 ± 10.2			76 ± 56	1.8 ± 1.1	27 ± 7.1
Dershowitz, 2023				15.8 ± 5.7	44.06 ± 13.59		37.76 ± 7.15	34.76 ± 8.64						
Fam, 2021	25			15.33 ± 3.89		50.8 ± 7.2		37 ± 11	58.3 ± 3.6				1.5 ± 0.4	28 ± 2
Hagemeyer, 2024	38					53.16 ± 6.28	42.33 ± 11	36.73 ± 9.04	55.66 ± 1.57			122.66 ± 84.9		24.1 ± 6.1
Hahn, 2020	28			11 ± 2	41 ± 19.46				53.66 ± 11.45	47 ± 4.6	9.66 ± 2.33	85 ± 31		
Lu, 2021	46	78.93 ± 26.63	187.6 ± 101.16			54.13 ± 9.64		37.23 ± 5.96	55.1 ± 9.43		10.4 ± 1.76	80.83 ± 24.41	2.26 ± 1.76	
Mao, 2022	15	81 ± 24.94	191.83 ± 96.92	13.5 ± 1.34		55.7 ± 20.2	42.6 ± 12.43	37.96 ± 8.58				70.1 ± 12.43	2.2 ± 1.63	
Ning, 2023	22		343.63 ± 222.84	14.8 ± 3.23	37 ± 9.8			45.9 ± 8.6	64.8 ± 9	44 ± 6		116.6 ± 47.5		40.86 ± 12.28
Wang, 2024	20		156.4 ± 27.8	18 ± 6	45.8 ± 2.6	56.2 ± 8.7	44.4 ± 5.4	34.3 ± 1.8	55 ± 4					34.3 ± 1.4
Webb, 2022	27				43.6 ± 11.8	50.7 ± 7.3	40 ± 7.4	37.6 ± 11.5	58.1 ± 8.5				1.5 ± 0.9	27.7 ± 5
Wei, 2022	30	68.33 ± 38.14	131.33 ± 81.73					46.5 ± 6.8	62.9 ± 8.8	42.5 ± 5.6				
Yu, 2022	17													
Hahn, 2019						54.4 ± 5.08	40.4 ± 3.92	35.4 ± 6.71						
Elgharably, 2019	4									45.25				
Mao, 2023	6	83.51 ± 6.22	190.78 ± 35.05			57.2 ± 6.24	44.68 ± 4.71	41.17 ± 2.86	47.5 ± 4.07			70.5 ± 2.88	6.33 ± 1.63	
Sun, 2021	6							35 ± 11.73				95 ± 17.7	4.08 ± 1.48	
Fam, 2024	20					54.2 ± 4.9				51.2 ± 4.6				
Lu, 2020	12													
Stoltz, 2023														
Weckbach, 2023	25													

Abbreviations: EDD, end‐diastolic diameter; EROA, effective regurgitant orifice area; FAC, fractional area change; IVC, inferior vena cava; LVEF, left ventricular ejection fraction; PASP, pulmonary arterial systolic pressure; PISA, proximal iso‐velocity surface area; RA, right atrium; RV, right ventricle; TAPSE, tricuspid annular plane systolic excursion; TR, tricuspid regurgitation; TV, tricuspid valve.

### 3.3 Echocardiographic Findings

**Figure 2:**
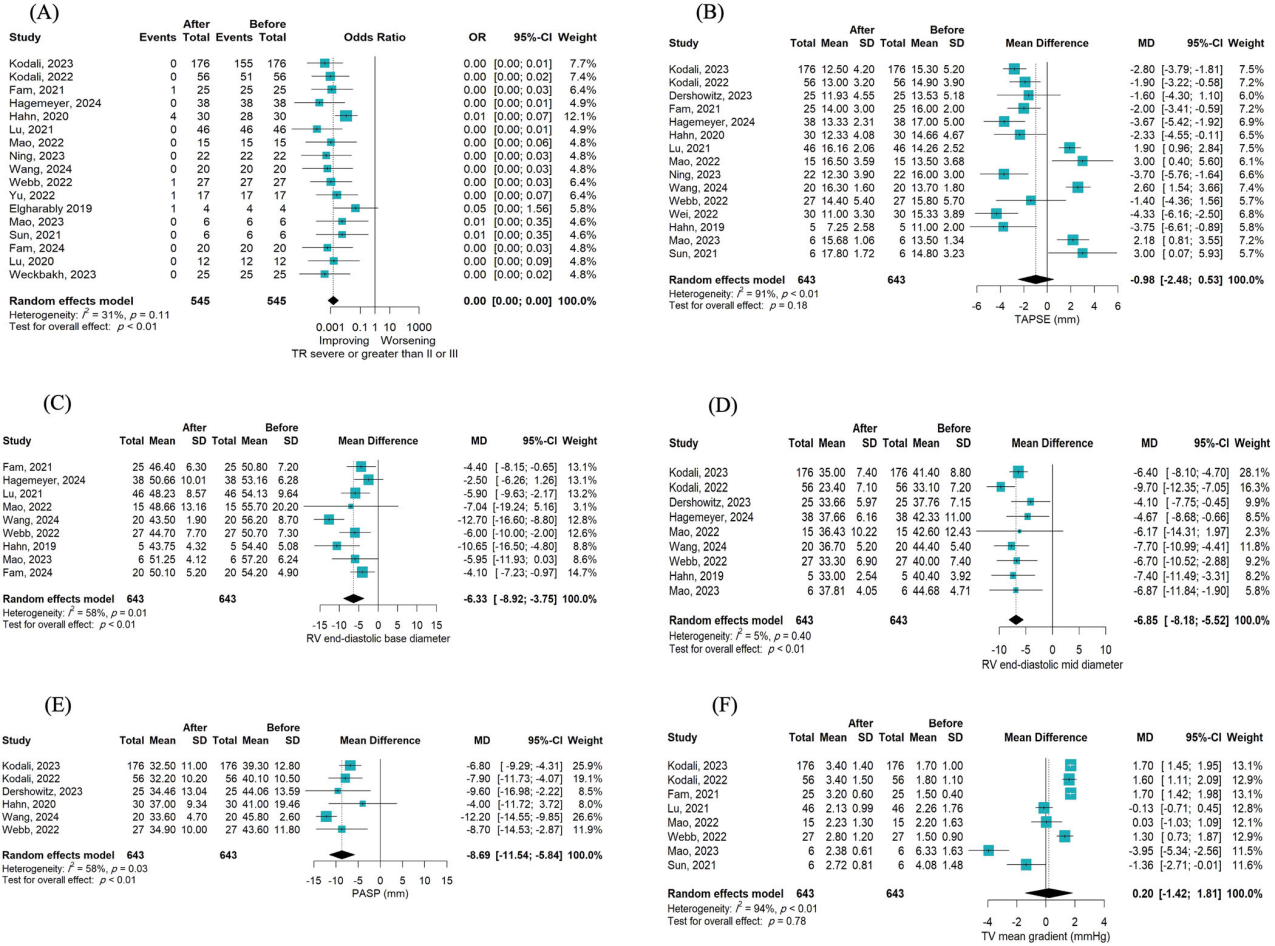
(A) Forest plot illustrating the odds ratio of severe TR in patients undergoing TTVR at baseline versus last follow‐up. (B) Forest plot illustrating the mean difference in TAPSE at baseline versus last follow‐up. (C) Forest plot illustrating the mean difference in RV end‐diastolic base diameter at baseline versus last follow‐up. (D) Forest plot illustrating the mean difference in RV end‐diastolic mid‐diameter at baseline versus last follow‐up. (E) Forest plot illustrating the mean difference in PASP at baseline versus last follow‐up. (F) Forest plot illustrating the mean difference in TV mean gradient at baseline versus last follow‐up. PASP, pulmonary arterial systolic pressure; RV, right ventricle; TAPSE, tricuspid annular plane systolic excursion; TR, tricuspid regurgitation; TV, tricuspid valve.

TTVR could significantly decrease the odds of grade ≥ 3 tricuspid regurgitation in the studied population at the latest follow‐up compared with baseline (OR = 0.0013; 95% CI [0.0006, 0.0027]; *p* < 0.001; I² = 31%). There was no significant change in the TV mean gradient at the final follow‐up compared to the baseline measurement (MD = 0.20; 95% CI [−1.42, 1.81]; *p* = 0.78; I² = 94%) [15, 23, 26, 27, 34, 37, 38, 39]. No significant change was observed in mean LVEF (MD = 0.96; 95% CI [−1.98, 3.90]; *p* = 0.47; I² = 65%) [15, 27, 28, 29, 37, 38, 39, 40] and in RV FAC at the last follow‐up compared to baseline (MD = −2.52; 95% CI [−6.9, 1.04]; *p* = 0.15; I² = 96%) [15, 23, 25, 26, 27, 29, 34, 37, 38, 39, 41]. No significant reduction in postprocedural TAPSE levels was demonstrated (MD = −0.98; 95% CI [−2.48, 0.53]; *p* = 0.18; I² = 91%) [15, 23, 24, 25, 26, 27, 28, 31, 34, 37, 38, 39, 40, 41]. A significant decline in PASP was observed at the last follow‐up compared to the baseline (MD = −8.69; 95% CI [−11.54, −8.54]; *p* < 0.001; I² = 58%) [15, 24, 28, 37, 38, 41]. There was a significant decrease in RV end‐diastolic base diameter (MD = −6.33 mm; 95% CI [−8.92, −3.75]; *p* < 0.001; I² = 58%) [15, 23, 25, 26, 27, 28, 31, 33, 39], and RV end‐diastolic mid diameter (MD = −6.33 mm; 95% CI [−8.18, −5.52]; *p* < 0.001; I² = 5%) [15, 25, 26, 27, 28, 31, 37, 38, 41], as well as a nonsignificant decrease in tricuspid annular diameter (MD = −7.51 mm; 95% CI [−18.73, −3.75]; *p* = 0.07; I² = 0%) [29, 40] at the last follow‐up compared to baseline. A significant reduction in RV (MD = −22.91; 95% CI [−29.64, −16.17]; *p* = 0.001; I² = 0%) [26, 27, 29, 39], RA (MD = −20.29; 95% CI [−31.85, −8.73]; *p* = 0.004; I² = 29%) [26, 27, 28, 29, 37, 38, 39, 40] volumes and IVC diameter (MD = −7.00; 95% CI [−9.85, −4.14]; *p* < 0.001; I² = 88%) [15, 23, 25, 28, 37, 38, 40] was observed following the procedure compared with the baseline (Figure 2, Supplementary Figure 2).

### 3.4 Clinical and Functional Improvements

**Figure 3:**
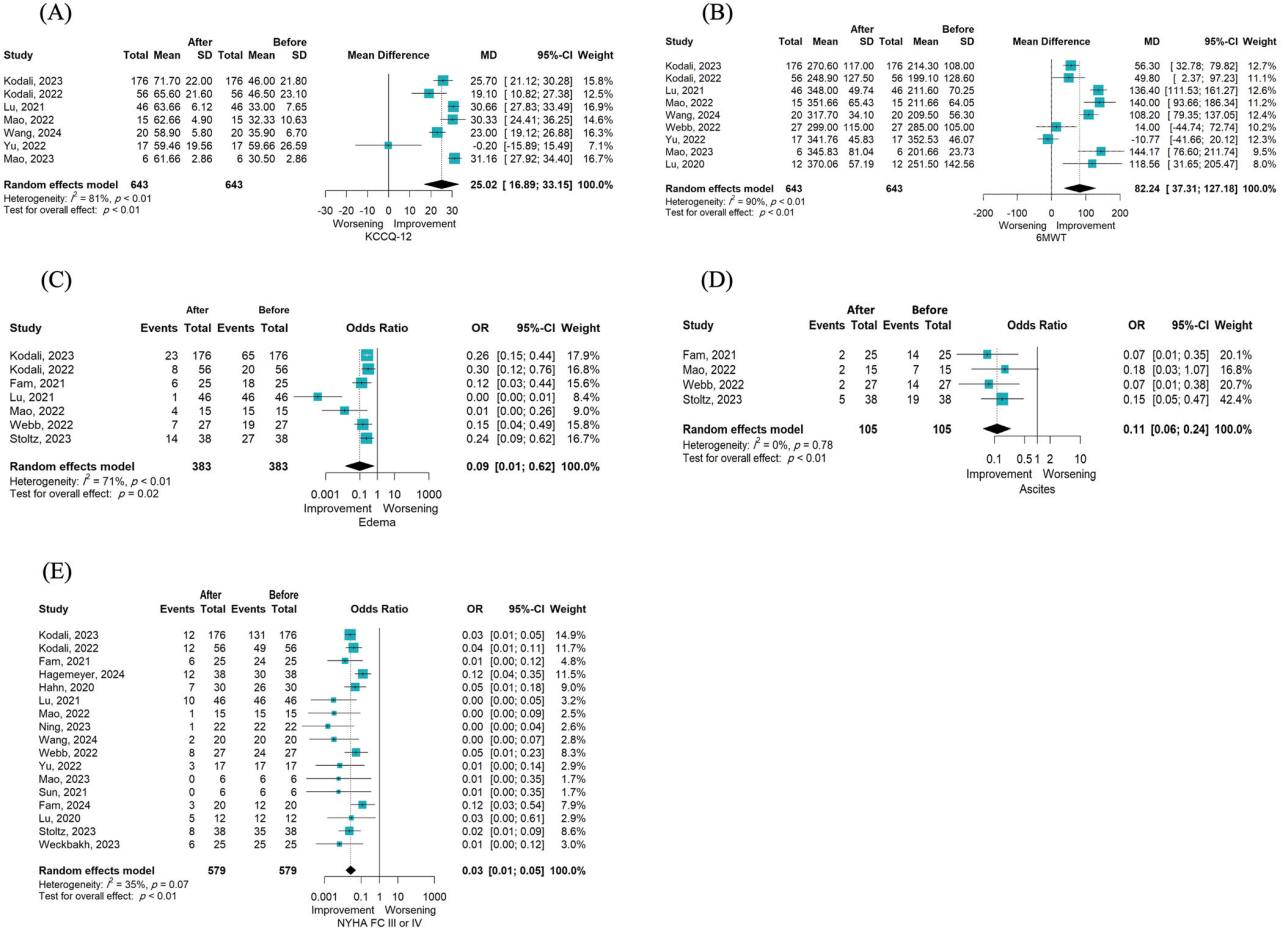
(A) Forest plot illustrating the mean difference in KCCQ‐12 at baseline versus last follow‐up. (B) Forest plot illustrating the mean difference in 6MWT at baseline versus last follow‐up. (C) Forest plot illustrating the odds ratio of edema at baseline versus last follow‐up. (D) Forest plot illustrating the odds ratio of ascites at baseline versus last follow‐up. (E) Forest plot illustrating the odds ratio of NYHA FC III or IV at baseline versus last follow‐up. 6MWT, 6‐min walk test; FC, functional class; KCCQ‐12, Kansas City Cardiomyopathy Questionnaire‐Short version; NYHA, New York Heart Association.

The odds of NYHA FC ≥ 3 (OR = 0.03; 95% CI [0.01, 0.05]; *p* < 0.001; I² = 35%) [15, 23, 24, 25, 26, 27, 28, 30, 33, 34, 35, 36, 37, 38, 39, 40, 42], edema (OR = 0.09; 95% CI [0.01, 0.62]; *p* = 0.02; I² = 71%) [15, 23, 26, 36, 37, 38, 39], and ascites (OR = 0.11; 95% CI [0.06, 0.24]; *p* = 0.002; I² = 0%) [15, 23, 26, 36] decreased significantly after TTVR compared with baseline. There was a significant increase in 6MWD (m) (MD = 82.24; 95% CI 37.31, 127.18]; *p* = 0.002; I² = 90%) [15, 26, 27, 28, 30, 35, 37, 38, 39] and Kansas City questionnaire (MD = 25.02; 95% CI [16.89, 33.15]; *p* < 0.001; I² = 81%) [26, 27, 28, 30, 37, 38, 39] at the last follow‐up compared to the baseline (Figure 3).

### 3.5 Major Adverse Cardiovascular Events (MACE)

**Figure 4:**
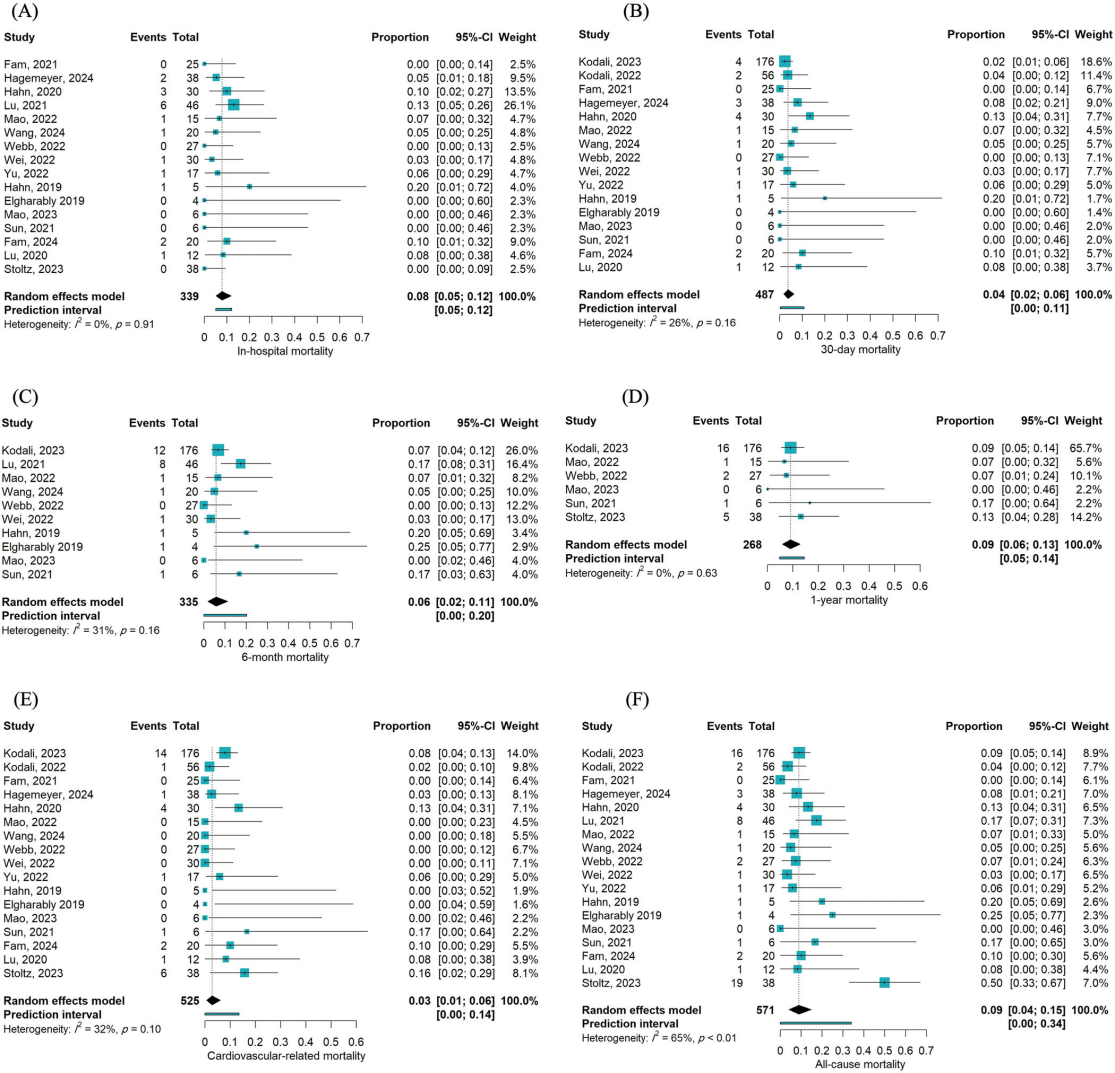
(A) Forest plot illustrating the proportion of in‐hospital mortality following TTVR. (B) Forest plot illustrating the proportion of 30‐day mortality following TTVR. (C) Forest plot illustrating the proportion of 6‐month mortality following TTVR. (D) Forest plot illustrating the proportion of one‐year mortality following TTVR. (E) Forest plot illustrating the proportion of cardiovascular‐related mortality following TTVR. (F) Forest plot illustrating the proportion of all‐cause mortality following TTVR.

**Figure 5:**
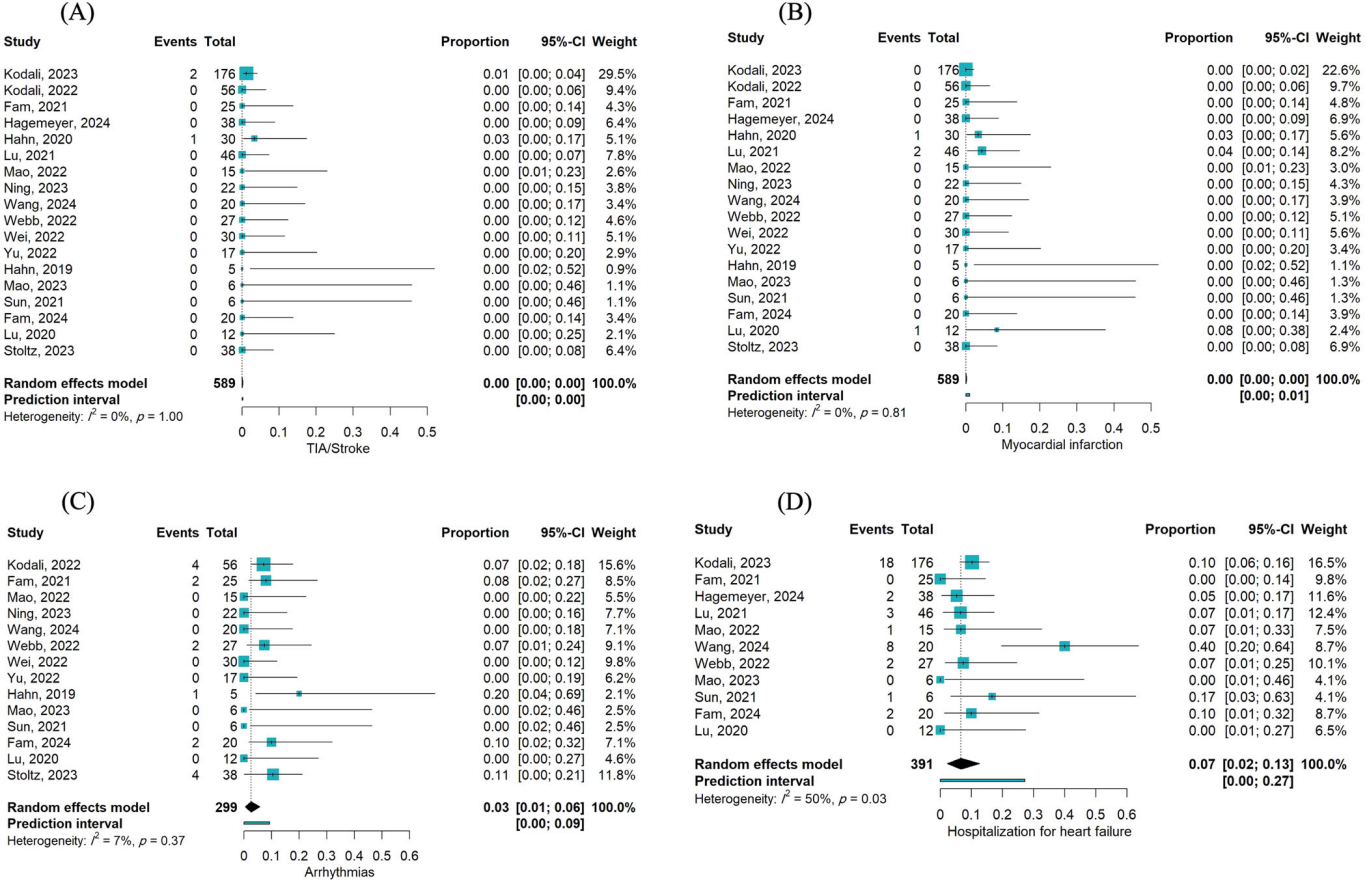
(A) Forest plot illustrating the proportion of TIA/stroke following TTVR. (B) Forest plot illustrating the proportion of myocardial infarction following TTVR. (C) Forest plot illustrating the proportion of arrhythmias following TTVR. (D) Forest plot illustrating the proportion of hospitalization due to heart failure following TTVR.

Eighteen studies reported mortality following TTVR. The pooled incidence of in‐hospital mortality was 8% (events/total = 19/339; 95% CI [0.05, 0.12]; I² = 0%) [15, 23, 24, 25, 26, 27, 28, 29, 30, 31, 32, 33, 34, 35, 36, 39], 30‐day mortality was 4% (Events/Total = 21/487; 95% CI [0.02, 0.06]; I² = 26%) [15, 23, 24, 25, 26, 27, 28, 29, 30, 31, 32, 33, 34, 35, 36, 39], 6‐month mortality was 6% (Events/Total = 26/335; 95% CI [0.02, 0.11]; I² = 31%) [15, 26, 27, 28, 29, 31, 32, 34, 38, 39], and 1‐year mortality was 9% (Events/Total = 25/268; 95% CI [0.06, 0.13]; I² = 0%) [15, 26, 27, 34, 36, 38]. The pooled incidence of cardiac mortality was 3% (Events/Total = 31/525; 95% CI [0.01, 0.06]; I² = 32%) [15, 23, 24, 25, 26, 27, 28, 29, 30, 31, 32, 33, 34, 35, 36, 37, 38] and all‐cause mortality was 9% (Events/Total = 64/571; 95% CI [0.04, 0.15]; I² = 65%) [15, 23, 24, 25, 26, 27, 28, 29, 30, 31, 32, 33, 34, 35, 36, 37, 38, 39] according to the last follow‐up (Figure 4). Arrhythmias was 3% (events/total = 15/299; 95% CI [0.01, 0.06]; I² = 7%) [15, 23, 26, 27, 28, 29, 30, 31, 33, 34, 35, 36, 37, 40], and Hospitalization due to HF was 7% (Events/Total = 37/391; 95% CI [0.02, 0.13]; I² = 50%) [15, 23, 25, 26, 27, 28, 33, 34, 35, 38, 39] at the last follow‐up after TTVR (Figure 5). Only a very small proportion of MI and TIA/stroke events were reported following TTVR at the last follow‐up.

### 3.6 Adverse Events

**Figure 6:**
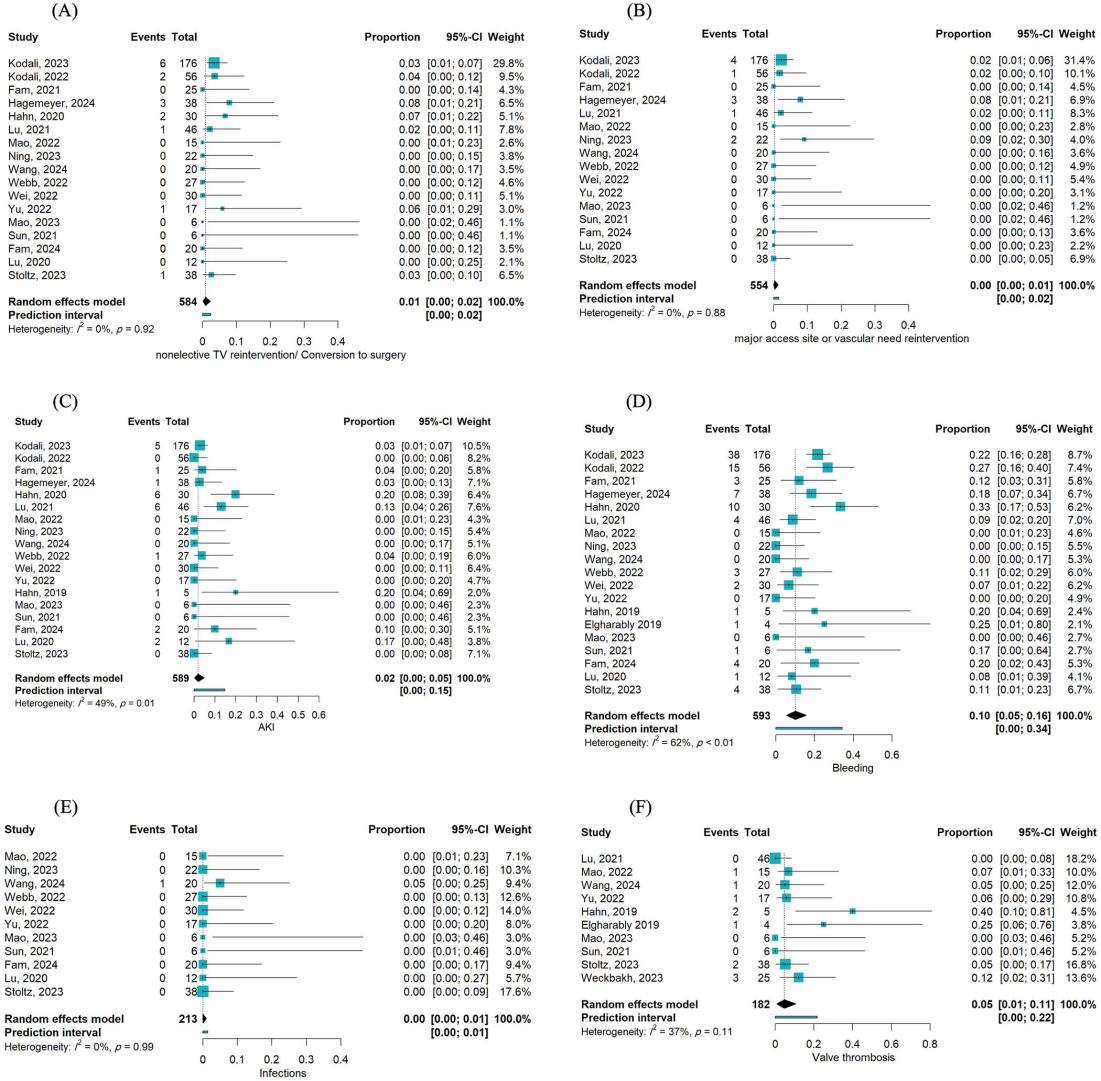
(A) Forest plot illustrating the proportion of non‐elective TV reintervention/conversion to surgery following TTVR. (B) Forest plot illustrating the proportion of major access site or vascular reintervention following TTVR. (C) Forest plot illustrating the proportion of AKI following TTVR. (D) Forest plot illustrating the proportion of bleeding following TTVR. (E) Forest plot illustrating the proportion of infection following TTVR. (F) Forest plot illustrating the proportion of valve thrombosis following TTVR. AKI, acute kidney injury; TV, tricuspid valve.

The pooled incidence of bleeding and AKI was 10% (95% CI [0.05, 0.16]) [15, 23, 24, 25, 26, 27, 28, 29, 30, 31, 32, 33, 34, 35, 36, 37, 38, 39, 40] and 2% (95% CI [0.00, 0.05]) [15, 23, 24, 25, 26, 27, 28, 29, 30, 31, 32, 33, 34, 35, 36, 37, 38], respectively, at the last follow‐up. Valve thrombosis occurred in 5% (95% CI [0.01, 0.11]) [26, 27, 28, 30, 31, 32, 34, 36, 39, 42], while device‐related pulmonary embolism was not reported. Major access site or vascular complications were also not reported. The incidence of device migration and non‐elective tricuspid valve reintervention or conversion to surgery was 1% each. Pulmonary complications, including pneumonia, pleural effusion, pneumothorax, atelectasis, and respiratory failure, were observed in 10% (95% CI [0.01, 0.11]) [26, 28, 29, 30, 32, 34, 39, 40], while infections were nearly absent. Detailed statistical results can be found in Figures 6, Supplementary Figure 3, and Supplementary Table 4.

### 3.7 Assessment of Risk of Bias and Heterogeneity

To assess the risk of bias in the included studies, we utilized the ROBINS‐I tool for observational studies and the JBI checklist for case series. Among the observational studies, two were found to have a low risk of bias, while the remaining studies had a medium risk of bias Supplementary Figure 4. For case series, all studies were rated as high quality, based on the JBI checklist Supplementary Figure 5. Given the significant heterogeneity observed in some analyses, we applied a random‐effects model to account for variability between the studies. This approach is suitable when there is substantial heterogeneity, as it provides a more flexible interpretation of the results by considering both within‐study and between‐study variation. We performed a leave‐one‐out sensitivity analysis for the major outcomes, testing the robustness of the results by sequentially removing each study and reanalyzing the data. This analysis revealed no substantial differences in the results, supporting the stability of our findings. These results are presented in Supplementary Tables 5–10.

### 3.8 Assessment of Publication Bias

NYHA functional class III or IV was subjected to publication bias as shown by the Egger's test (*p* = 0.015). After adding three studies using the trim‐and‐fill method, the change remained significant (OR = 0.03; 95% CI [0.02, 0.07]; *p* < 0.001; I² = 42%). For the incidence of MI following TTVR, Egger's test had a p‐value of 0.006. After adjusting by adding nine studies, the proportion remained the same. The p‐value for device‐related pulmonary embolism was < 0.001. After adjustment by adding six studies using the trim‐and‐fill method, the proportion did not change. Other measures, including clinical outcomes and echocardiographic measures, were insignificant and showed symmetrical distribution, as illustrated in the funnel plots (Supplementary Figures 6–11).

## 4 Discussion

**Figure 7:**
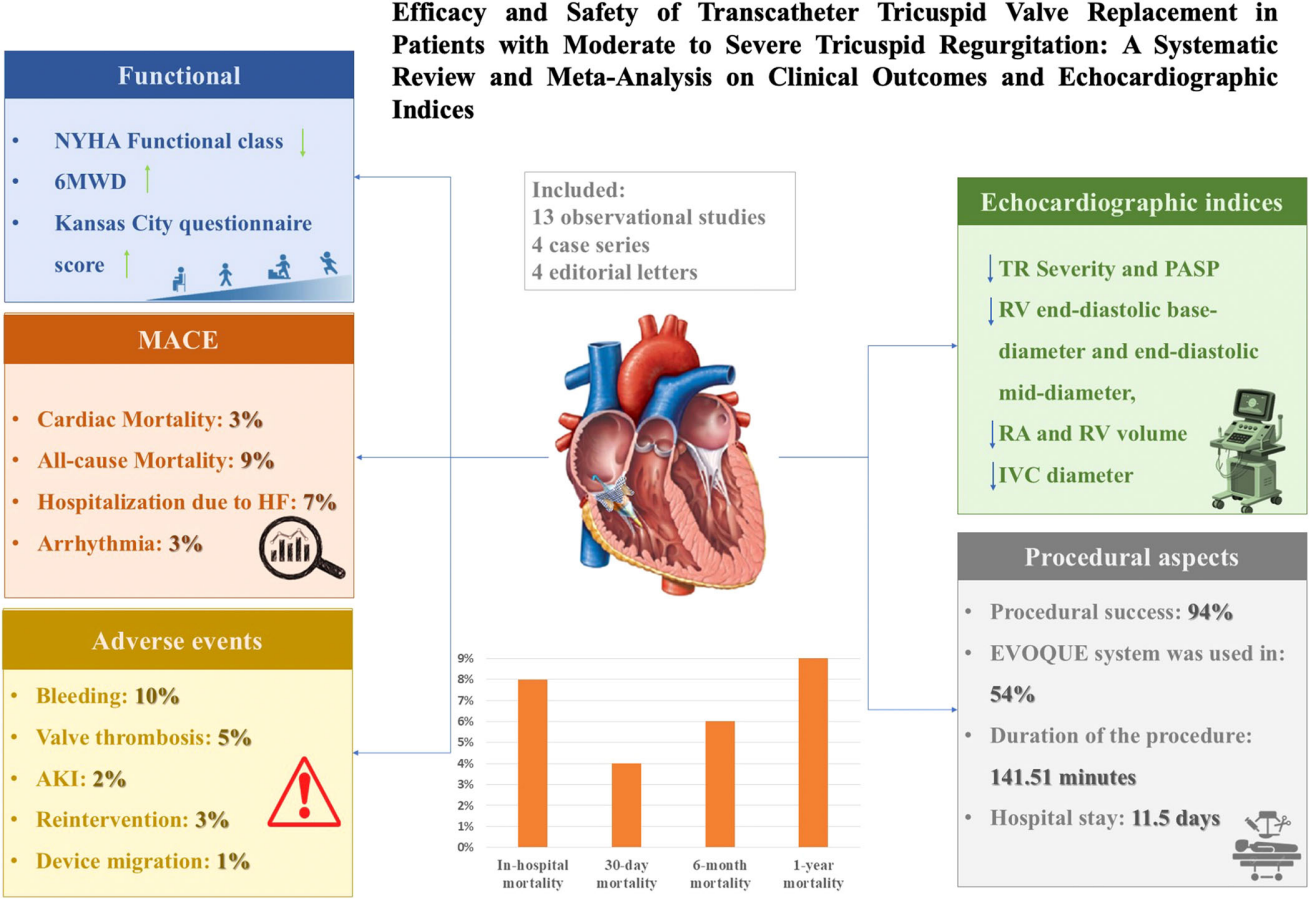
Graphical abstract summarizing the study's key findings.

TTVR is a promising, less invasive alternative to surgical treatments for tricuspid valve disease. This pooled analysis assessed the effectiveness and safety of TTVR, and the key findings are as follows: 1. TTVR is a procedure with a high success rate that can significantly improve clinical and functional outcomes. 2. TTVR was associated with lower mortality, morbidity, and complication rates compared to surgical options, making it a relatively safe procedure. Despite these successes, challenges remain. Figure 7 depicts the graphical abstract summarizing the key findings and concepts of the study.

Despite its prevalence, TR has not received adequate attention due to several factors. One reason is the longstanding belief that treating left‐sided valve diseases would also effectively address TR [43]. This misconception has resulted in isolated tricuspid valve disease becoming a neglected area in many interventional cardiology circles [44]. Another reason is the high operative mortality rates of about 10% [45, 46] associated with isolated TV repair/replacement surgeries, which are often perceived as high‐risk and low‐reward by many medical professionals. This concern is compounded by the fact that many patients with TR present with comorbidities such as atrial fibrillation [47], renal diseases, and heart failure [48], making surgery more complicated and contributing to a reported 44% mortality rate over the 10 years following surgical procedures [49]. In contrast, despite most patients having serious conditions, the 1‐year mortality rate after transcatheter intervention was only about 14%, suggesting that refined nonsurgical techniques, such as transcatheter interventions, could lead to better patient outcomes [50, 51].

This highlights the growing potential of novel nonsurgical methods, such as TTVR and transcatheter edge‐to‐edge repair (TEER), which were once considered last‐resort options after surgical failure but are now emerging as promising primary treatment approaches for TR. While TTVR is the focus of this study, TEER, as demonstrated in recent meta‐analyses [52, 53], has also shown significant improvements in TR severity and clinical outcomes, including reductions in tricuspid valve volume and TR grade, as well as enhancements in NYHA class and 6‐min walking distance. However, anatomical challenges—such as large tricuspid annulus dimensions (> 40 mm), multiple leaflets, a nonplanar, elliptical shape, complex right ventricular morphology (including trabeculations, muscle bands, and a thin apical wall), and coaptation defects like large gaps or leaflet tethering—can significantly affect procedural success [54, 55]. These complex features not only demand careful case selection but also tend to favor TTVR over TEER, as TTVR has demonstrated greater effectiveness in eliminating severe TR in patients with such anatomies [56, 57, 58]. Additionally, residual TR after T‐TEER is linked to worse clinical outcomes, highlighting the potential advantage of TTVR for achieving more durable TR reduction [59, 60]. Consequently, TTVR may offer a more suitable option for patients with complex anatomical characteristics.

The procedural success rate in this study was 97%, which is higher than the 92% reported in meta‐analyses of TTVR studies [61] and comparable to the 97% success rate observed in meta‐analyses evaluating TEER [62]. The analysis of MACE indicated a pooled incidence of in‐hospital mortality at 8%, with rates observed at 30 days at 4% and 6 months at 6%, and with a 1‐year mortality rate of 9%. These rates compare favorably to the anticipated outcomes for untreated severe TR in similar patient populations. This 1‐year mortality rate is also notably lower compared to the 10% rate reported in previous meta‐analyses, which included smaller populations of patients [19]. The 30‐day mortality rate was 6% in the meta‐analysis of TEER by Siddiqui et al. [53]. In the study by Khalid et al. [63], the 1‐year mortality rate was 14% among patients undergoing transcatheter tricuspid valve repair and replacement. Similarly, Buğan et al. [61] reported a 10% 1‐year mortality in TTVR studies, and Rehan et al. [62] observed a similar 10% rate in TEER studies. These findings suggest promising trends in reducing short‐term and midterm mortality rates in more recent TTVR trials. Almost all procedural complications, including valve thrombosis, device migration, major access site complications, or the need for reintervention, were negligible. However, slight chances of bleeding and post‐op pulmonary complications necessitate closer monitoring for these incidents. Although lifelong anticoagulation with either vitamin K antagonists or direct oral anticoagulants is recommended due to the high thrombotic risk associated with the low‐flow tricuspid position [64], its use must be carefully managed, as major bleeding has been reported in 10–27% of patients [38, 64].

Echocardiographic assessments revealed a significant reduction in the severity of TR at follow‐up, with a marked decrease in the number of patients experiencing grade ≥ 3 TR. Moreover, studies have shown that even a small improvement in TR can yield significant results and prevent further deteriorations such as recurrent tricuspid regurgitation, severe right ventricular failure, and liver congestion [65]. Additionally, there was a significant decline in PASP and RV dimensions postprocedure, indicating improved hemodynamics. However, certain echocardiographic parameters, including LVEF, tricuspid valve mean gradient, RV FAC, and TAPSE, showed no significant improvement. The early and abrupt elimination of TR is believed to increase RV afterload, which could contribute to the lack of improvement in TAPSE and RV FAC [66, 67]. This concept aligns with the importance of RV–pulmonary artery coupling, as reflected by the TAPSE/sPAP ratio; impaired (uncoupled) states may heighten the risk of acute RV failure, particularly in patients with severe baseline RV dysfunction or pulmonary hypertension (pulmonary vascular resistance > 4 Wood units) [68]. A possible reason for the absence of significant change in LVEF is that TR predominantly affects right heart function and thus has a limited direct impact on left heart performance [52]. These findings are consistent with a recent systematic review and meta‐analysis by Rehan et al. [52], which found a similar lack of significant change in these parameters.

Patients exhibited significant functional improvements following TTVR. There was a notable reduction in the number of patients classified as NYHA functional class ≥ 3, as well as improvements in 6MWD and quality of life as measured by the Kansas City Cardiomyopathy Questionnaire. These results were in line with previous systematic reviews on TTVR and TEER [19, 62, 69], which further validates that TTVR can significantly improve patient lives. While interstudy variability in NYHA classification exists, the concordance of improvements across subjective (NYHA, KCCQ) and objective (6MWD) endpoints reinforces the robustness of these findings. There was a significant decrease in the incidence of edema and ascites. These findings suggest effective management of right‐sided heart failure symptoms, corroborating the reduction in right atrial volume and right ventricular end‐diastolic diameter observed in other studies [70, 71].

To our knowledge, this is the first systematic review and meta‐analysis focusing exclusively on TTVR for TR, providing a targeted evaluation of its effectiveness, safety, and procedural success. Unlike prior analyses that combined repair and replacement procedures, our study isolates TTVR, offering clearer insights into this specific intervention. Including a wide range of clinical, echocardiographic, and procedural outcomes, minimal evidence of publication bias, and relatively consistent follow‐up durations across studies further strengthen the validity of our findings. However, several limitations should be acknowledged. Most included studies were single‐arm and retrospective, increasing susceptibility to bias and limiting causal inference. Variability in patient characteristics, procedural techniques, devices, endpoints, and follow‐up durations contributed to heterogeneity, complicating direct comparisons. The use of different TTVR devices, each with distinct implantation mechanisms, may also have influenced outcomes. Moreover, the patient population largely consisted of individuals with severe comorbidities and high surgical risk, limiting generalizability to broader populations. Moving forward, standardized definitions, consistent endpoints, and the adoption of criteria such as those proposed by the Valve Academic Research Consortium (VARC) for transcatheter aortic valve replacement (TAVR) will be critical to improving comparability and reliability in TTVR studies [72]. As devices evolve and operator experience increases, further improvements in outcomes are anticipated.

## 5 Conclusion

TTVR emerges as a promising alternative for patients with severe TR who are not candidates for surgery. This pooled analysis highlights the procedure's potential, demonstrating a low incidence of mortality and MACE, alongside notable improvements in clinical outcomes, echocardiographic parameters, and quality of life. Furthermore, complications associated with the procedure were minimal. While transcatheter repair of the tricuspid valve is a well‐established approach, there are cases where valve replacement becomes necessary. TTVR devices are particularly advantageous compared to repair methods because they are not dependent on valve leaflet condition or the underlying cause of TR, contributing to a lower mortality risk. Moreover, in an era with a growing array of available devices, it is essential to identify the most effective technology to optimize patient outcomes. Therefore, future large‐scale randomized controlled trials should focus on comparing transcatheter tricuspid valve repair with replacement and evaluating different device systems for TTVR to establish the most effective treatment strategy.

## Author Contributions

**Pouria Azami:** conceptualization, writing – original draft, writing – review and editing, methodology, project administration, visualization. **Alireza Hosseinpour:** methodology, writing – review and editing, formal analysis, writing – original draft. **Jahangir Kamalpour:** writing – original draft, writing – review and editing, formal analysis. **Farshad Rajabi:** data curation, software, validation, writing – review and editing. **Iman Razeghian‐Jahromi:** visualization, methodology, conceptualization. **Sarvenaz Farhangdoost:** data curation, software, validation. **Reza Golchin Vafa:** data curation, software, validation. **Ghazaleh Bagheri:** data curation, software.

## Ethics Statement

The authors have nothing to report.

## Conflicts of Interest

The authors declare no known competing financial interests or personal relationships that could have influenced the work reported in this paper.

## Transparency Statement

The lead author Pouria Azami affirms that this manuscript is an honest, accurate, and transparent account of the study being reported; that no important aspects of the study have been omitted; and that any discrepancies from the study as planned (and, if relevant, registered) have been explained.

## Supporting information

Supplementary materials.

## Data Availability

The data that support the findings of this study are available from the corresponding author upon reasonable request.
