# Supplementary material for: Efficacy and Safety of Transcatheter Tricuspid Valve Replacement in Patients With Moderate to Severe Tricuspid Regurgitation: A Systematic Review and Meta‐Analysis on Clinical Outcomes and Echocardiographic Indices
Source: Health Sci Rep. 2025 Jun 23;8(6):e70950. doi: 10.1002/hsr2.70950 (PMC12183386; doi:10.1002/hsr2.70950)
Supplement: Supplementary file 1 — Supplementary materials. [file HSR2-8-e70950-s001.docx]

**Supplementary materials:**

**Supplementary Table 1:** Displays the detailed search strategy used in the study.

| **Database** | **Search Strategy** | **Articles retrieved** |
| --- | --- | --- |
| PubMed | ("transcatheter"[All Fields] AND ("tricuspid"[All Fields] OR "tricuspidal"[All Fields] OR "tricuspidization"[All Fields]) AND ("replace"[All Fields] OR "replaceable"[All Fields] OR "replaced"[All Fields] OR "replaces"[All Fields] OR "replacing"[All Fields] OR "replacment"[All Fields] OR "replantation"[MeSH Terms] OR "replantation"[All Fields] OR "replacement"[All Fields] OR "replacements"[All Fields])) OR ("transcatheter"[All Fields] AND ("tricuspid"[All Fields] OR "tricuspidal"[All Fields] OR "tricuspidization"[All Fields]) AND ("intervention s"[All Fields] OR "interventions"[All Fields] OR "interventive"[All Fields] OR "methods"[MeSH Terms] OR "methods"[All Fields] OR "intervention"[All Fields] OR "interventional"[All Fields])) OR ("transcatheter"[All Fields] AND ("tricuspid"[All Fields] OR "tricuspidal"[All Fields] OR "tricuspidization"[All Fields]) AND ("drug implants"[MeSH Terms] OR ("drug"[All Fields] AND "implants"[All Fields]) OR "drug implants"[All Fields] OR "implant"[All Fields] OR "embryo implantation"[MeSH Terms] OR ("embryo"[All Fields] AND "implantation"[All Fields]) OR "embryo implantation"[All Fields] OR "implantation"[All Fields] OR "implant s"[All Fields] OR "implantability"[All Fields] OR "implantable"[All Fields] OR "implantables"[All Fields] OR "implantate"[All Fields] OR "implantated"[All Fields] OR "implantates"[All Fields] OR "implantations"[All Fields] OR "implanted"[All Fields] OR "implanter"[All Fields] OR "implanters"[All Fields] OR "implanting"[All Fields] OR "implantion"[All Fields] OR "implantitis"[All Fields] OR "implants"[All Fields])) OR ("transcatheter"[All Fields] AND ("tricuspid valve"[MeSH Terms] OR ("tricuspid"[All Fields] AND "valve"[All Fields]) OR "tricuspid valve"[All Fields]) AND ("drug implants"[MeSH Terms] OR ("drug"[All Fields] AND "implants"[All Fields]) OR "drug implants"[All Fields] OR "implant"[All Fields] OR "embryo implantation"[MeSH Terms] OR ("embryo"[All Fields] AND "implantation"[All Fields]) OR "embryo implantation"[All Fields] OR "implantation"[All Fields] OR "implant s"[All Fields] OR "implantability"[All Fields] OR "implantable"[All Fields] OR "implantables"[All Fields] OR "implantate"[All Fields] OR "implantated"[All Fields] OR "implantates"[All Fields] OR "implantations"[All Fields] OR "implanted"[All Fields] OR "implanter"[All Fields] OR "implanters"[All Fields] OR "implanting"[All Fields] OR "implantion"[All Fields] OR "implantitis"[All Fields] OR "implants"[All Fields])) OR ("transcatheter"[All Fields] AND ("tricuspid valve"[MeSH Terms] OR ("tricuspid"[All Fields] AND "valve"[All Fields]) OR "tricuspid valve"[All Fields]) AND ("intervention s"[All Fields] OR "interventions"[All Fields] OR "interventive"[All Fields] OR "methods"[MeSH Terms] OR "methods"[All Fields] OR "intervention"[All Fields] OR "interventional"[All Fields])) OR ("transcatheter"[All Fields] AND ("tricuspid valve"[MeSH Terms] OR ("tricuspid"[All Fields] AND "valve"[All Fields]) OR "tricuspid valve"[All Fields]) AND ("replace"[All Fields] OR "replaceable"[All Fields] OR "replaced"[All Fields] OR "replaces"[All Fields] OR "replacing"[All Fields] OR "replacment"[All Fields] OR "replantation"[MeSH Terms] OR "replantation"[All Fields] OR "replacement"[All Fields] OR "replacements"[All Fields])) OR "TTVR"[All Fields] OR "TTVI"[All Fields] | 1907 |
| Embase | 'transcatheter tricuspid valve replacement'/exp OR 'transcatheter tricuspid valve replacement' OR 'transcatheter tricuspid valve intervention'/exp OR 'transcatheter tricuspid valve intervention' OR 'transcatheter tricuspid valve implantation'/exp OR 'transcatheter tricuspid valve implantation' OR 'transcatheter tricuspid replacement' OR 'transcatheter tricuspid intervention' OR 'transcatheter tricuspid implantation' OR 'tricuspid implantation':ti,ab,kw OR 'tricuspid replacement':ti,ab,kw OR 'tricuspid intervention':ti,ab,kw OR ttvr OR ttvi | 630 |
| Scopus | ( ALL ( "transcatheter tricuspid valve intervention" ) ) OR ( ALL ( "transcatheter tricuspid valve replacement" ) ) OR ( ALL ( "transcatheter tricuspid valve implantation" ) ) OR ( ALL ( "transcatheter tricuspid implantation" ) ) OR ( ALL ( "transcatheter tricuspid replacement" ) ) OR ( ALL ( "transcatheter tricuspid intervention" ) ) OR ( ALL ( "tricuspid intervention" ) ) OR ( ALL ( "tricuspid implantation" ) ) OR ( ALL ( "tricuspid replacement" ) ) OR ( ALL ( ttvi ) ) OR ( ALL ( ttvr ) ) | 1445 |
| Web of Science | transcatheter tricuspid valve replacement (All Fields) or transcatheter tricuspid valve intervention (All Fields) or transcatheter tricuspid valve implantation (All Fields) or transcatheter tricuspid implantation (All Fields) or transcatheter tricuspid replacement (All Fields) or transcatheter tricuspid intervention (All Fields) or TTVR (All Fields) or TTVI (All Fields) | 1603 |
| Google Scholer | intitle:("transcatheter tricuspid valve replacement" OR "transcatheter tricuspid valve implantation" OR TTVR OR "tricuspid replacement" OR "tricuspid implantation") AND ("clinical outcomes" OR "echocardiographic outcomes" OR "major adverse cardiovascular events" OR "events" OR "outcomes") | 1810 |

**Supplementary Table 2:** Presents the PRISMA checklist for systematic reviews and meta-analyses.

| **Section and Topic** | **Item #** | **Checklist item** | **Location where item is reported** |
| --- | --- | --- | --- |
| **TITLE** | | |  |
| Title | 1 | Identify the report as a systematic review. | 1 |
| **ABSTRACT** | | |  |
| Abstract | 2 | See the PRISMA 2020 for Abstracts checklist. | 2 |
| **INTRODUCTION** | | |  |
| Rationale | 3 | Describe the rationale for the review in the context of existing knowledge. | 3 |
| Objectives | 4 | Provide an explicit statement of the objective(s) or question(s) the review addresses. | 4 |
| **METHODS** | | |  |
| Eligibility criteria | 5 | Specify the inclusion and exclusion criteria for the review and how studies were grouped for the syntheses. | 5 |
| Information sources | 6 | Specify all databases, registers, websites, organisations, reference lists and other sources searched or consulted to identify studies. Specify the date when each source was last searched or consulted. | 4 |
| Search strategy | 7 | Present the full search strategies for all databases, registers and websites, including any filters and limits used. | 4 |
| Selection process | 8 | Specify the methods used to decide whether a study met the inclusion criteria of the review, including how many reviewers screened each record and each report retrieved, whether they worked independently, and if applicable, details of automation tools used in the process. | 5 |
| Data collection process | 9 | Specify the methods used to collect data from reports, including how many reviewers collected data from each report, whether they worked independently, any processes for obtaining or confirming data from study investigators, and if applicable, details of automation tools used in the process. | 6 |
| Data items | 10a | List and define all outcomes for which data were sought. Specify whether all results that were compatible with each outcome domain in each study were sought (e.g. for all measures, time points, analyses), and if not, the methods used to decide which results to collect. | 5, 6 |
|  | 10b | List and define all other variables for which data were sought (e.g. participant and intervention characteristics, funding sources). Describe any assumptions made about any missing or unclear information. | 5, 6 |
| Study risk of bias assessment | 11 | Specify the methods used to assess risk of bias in the included studies, including details of the tool(s) used, how many reviewers assessed each study and whether they worked independently, and if applicable, details of automation tools used in the process. | 6, 7 |
| Effect measures | 12 | Specify for each outcome the effect measure(s) (e.g. risk ratio, mean difference) used in the synthesis or presentation of results. | 6, 7 |
| Synthesis methods | 13a | Describe the processes used to decide which studies were eligible for each synthesis (e.g. tabulating the study intervention characteristics and comparing against the planned groups for each synthesis (item #5)). | 5 |
|  | 13b | Describe any methods required to prepare the data for presentation or synthesis, such as handling of missing summary statistics, or data conversions. | 5, 6 |
|  | 13c | Describe any methods used to tabulate or visually display results of individual studies and syntheses. | 5 |
|  | 13d | Describe any methods used to synthesize results and provide a rationale for the choice(s). If meta-analysis was performed, describe the model(s), method(s) to identify the presence and extent of statistical heterogeneity, and software package(s) used. | 5 |
|  | 13e | Describe any methods used to explore possible causes of heterogeneity among study results (e.g. subgroup analysis, meta-regression). | 6, 7 |
|  | 13f | Describe any sensitivity analyses conducted to assess robustness of the synthesized results. | 6, 7 |
| Reporting bias assessment | 14 | Describe any methods used to assess risk of bias due to missing results in a synthesis (arising from reporting biases). | 6, 7 |
| Certainty assessment | 15 | Describe any methods used to assess certainty (or confidence) in the body of evidence for an outcome. | 6, 7 |
| **RESULTS** | | |  |
| Study selection | 16a | Describe the results of the search and selection process, from the number of records identified in the search to the number of studies included in the review, ideally using a flow diagram. | 7 |
|  | 16b | Cite studies that might appear to meet the inclusion criteria, but which were excluded, and explain why they were excluded. | 7, 27 |
| Study characteristics | 17 | Cite each included study and present its characteristics. | 7, 27 |
| Risk of bias in studies | 18 | Present assessments of risk of bias for each included study. | 10 |
| Results of individual studies | 19 | For all outcomes, present, for each study: (a) summary statistics for each group (where appropriate) and (b) an effect estimate and its precision (e.g. confidence/credible interval), ideally using structured tables or plots. | 8, 9, 10 |
| Results of syntheses | 20a | For each synthesis, briefly summarise the characteristics and risk of bias among contributing studies. | 8, 9, 10 |
|  | 20b | Present results of all statistical syntheses conducted. If meta-analysis was done, present for each the summary estimate and its precision (e.g. confidence/credible interval) and measures of statistical heterogeneity. If comparing groups, describe the direction of the effect. | 8, 9 |
|  | 20c | Present results of all investigations of possible causes of heterogeneity among study results. | 10 |
|  | 20d | Present results of all sensitivity analyses conducted to assess the robustness of the synthesized results. | 10 |
| Reporting biases | 21 | Present assessments of risk of bias due to missing results (arising from reporting biases) for each synthesis assessed. | 10 |
| Certainty of evidence | 22 | Present assessments of certainty (or confidence) in the body of evidence for each outcome assessed. | 10 |
| **DISCUSSION** | | |  |
| Discussion | 23a | Provide a general interpretation of the results in the context of other evidence. | 11 |
|  | 23b | Discuss any limitations of the evidence included in the review. | 14 |
|  | 23c | Discuss any limitations of the review processes used. | 14 |
|  | 23d | Discuss implications of the results for practice, policy, and future research. | 15 |
| **OTHER INFORMATION** | | |  |
| Registration and protocol | 24a | Provide registration information for the review, including register name and registration number, or state that the review was not registered. | 4 |
|  | 24b | Indicate where the review protocol can be accessed, or state that a protocol was not prepared. | 4 |
|  | 24c | Describe and explain any amendments to information provided at registration or in the protocol. | - |
| Support | 25 | Describe sources of financial or non-financial support for the review, and the role of the funders or sponsors in the review. | 15 |
| Competing interests | 26 | Declare any competing interests of review authors. | 16 |
| Availability of data, code and other materials | 27 | Report which of the following are publicly available and where they can be found: template data collection forms; data extracted from included studies; data used for all analyses; analytic code; any other materials used in the review. | - |

**Supplementary Table 3:** Additional Baseline Characteristics.

| First Author, year | Hb | | WBC | PLT |  | BNP | PT | INR | Uric acid | NT-PRO BNP | Albumin | AST | ALT | ALP | Cr | GFR | STS  score (%) | Malignancy | Severe liver  disease | Prior hospitalization due to HF | Prior  gastrointestinal  bleeding |
| --- | --- | --- | --- | --- | --- | --- | --- | --- | --- | --- | --- | --- | --- | --- | --- | --- | --- | --- | --- | --- | --- |
| Kodali, 2023 |  | |  |  |  | 367±292 | 17.73±5.83 | 1.53±0.59 | 7.16±2.91 | 1644±1138 | 3.96±0.37 | 27±11.21 | 18.66±8.97 | 112.33±48.59 | 1.13±0.37 | 50.5±15.32 | 7.4±5.8 | 50 | 23 | 72 | 29 |
| Kodali, 2022 |  | |  |  |  | 678±1813 | 18.8±8.2 | 1.6±0.74 | 7.4±2.9 | 678.8±1813.8 | 4±0.6 | 29±10.1 | 22.2±11.7 | 115.7±62.5 | 1.2±0.3 | 49±12.9 | 7.7±5.3 |  |  | 20 | 11 |
| Dershowitz, 2023 |  | |  |  |  |  |  |  |  |  |  |  |  |  |  |  |  |  |  |  |  |
| Fam, 2021 |  | |  |  |  |  |  |  |  | 3106±2028 |  | 30±4 | 25±10 |  |  | 52±8 | 9.1±2.3 |  |  |  |  |
| Hagemeyer, 2024 | |  |  |  |  |  |  |  |  | 1745±2291 | 3.91±0.51 |  | 17.33±6.93 |  | 1.24±0.50 | 47.66±16.94 |  |  |  |  |  |
| Hahn, 2020 | 10.8±2.56 | |  |  |  |  |  |  |  | 1897±1609 | 3.6±0.62 | 31±11.67 | 22.66±7.78 | 101.66±52.93 | 1.45±0.91 | 46.33±20.23 |  |  | 6 | 17 |  |
| Lu, 2021 | 10.12±2.20 | |  |  |  | 212±215 |  |  |  | 839.66±723 | 3.76±0.54 |  |  |  | 0.93±0.23 | 55.03±16.59 |  | 3 | 15 |  | 12 |
| Mao, 2022 | 10.4±2.24 | |  |  |  | 217+200 |  |  |  | 856±589 | 3.6±0.81 | 28.9±19.05 | 17.56±12.10 |  | 1±0.49 | 56.56±21.75 | 10.3±0.43 |  | 5 |  | 4 |
| Ning, 2023 |  | |  |  |  |  |  |  |  | 761±717 | 4.07±0.29 |  |  |  |  |  |  | 2 |  |  |  |
| Wang, 2024 |  | |  |  |  |  |  |  |  | 1480±628 |  | 29.1±8.8 | 24.1±10.7 |  | 1.34±0.26 | 56.2±10.5 | 11.3±1.9 |  | 6 |  | 6 |
| Webb, 2022 |  | |  |  |  |  |  |  |  | 2303±445 |  | 28±12 | 20±12 |  |  | 52±20 | 8.6±6.5 |  |  |  |  |
| Wei, 2022 | 12.03±2.10 | | 4.77±1.88 | 144.3±40.8 |  | 191±158 |  |  |  |  | 4.13±0.36 |  |  |  | 0.87±0.28 |  | 8.96±4.96 |  |  |  |  |
| Yu, 2022 |  | |  |  |  |  |  |  |  |  |  |  |  |  |  |  |  |  |  |  |  |
| Hahn, 2019 | 9.8±2.4 | |  |  |  |  |  |  |  | 3084±3228 | 3.5±0.74 | 33.2±10.52 | 13.6±7.09 | 186.2±210.2 |  | 52.6±16.63 |  | 1 | 1 |  |  |
| Elgharably 2019 |  | |  |  |  |  |  |  |  |  |  |  |  |  |  |  |  |  |  |  |  |
| Mao, 2023 | 9.97±5.51 | |  |  |  | 236±79 |  | 1/35±0/41 |  | 815± 157 | 3.5±0.27 | 29.85±8.67 | 18.4±4.72 |  | 1.01±0.15 | 53.63±6.10 | 10.11±1.67 | 1 | 3 |  | 0 |
| Sun, 2021 |  | |  |  |  |  |  |  |  |  |  |  |  |  |  |  | 9.75±1.66 |  |  |  |  |
| Fam, 2024 |  | |  |  |  |  |  |  |  |  |  |  |  |  |  |  | 6.2±3.9 |  |  |  |  |

**Supplementary Table 4:** Clinical Outcome Incidence and Echocardiographic Parameter Changes Following TTVR.

| **Incidence Metrics** | **NO. of  Included articles** | **Pooled  incidence (%)** | **Event/Total** | **95% CI** | **I2%** | **P value for heterogenicity** |
| --- | --- | --- | --- | --- | --- | --- |
| Technical success | 20 | 94 | 592/618 | [0.91; 0.96] | 0 | 0.92 |
| Cardiac mortality | 17 | 3 | 31/525 | [0.01; 0.06] | 32 | 0.1 |
| All-cause mortality | 18 | 9 | 64/571 | [0.04; 0.15] | 65 | <0.01 |
| MI | 18 | 0 | 4/589 | [0.00; 0.00] | 0 | 0.81 |
| Arrhythmias | 14 | 3 | 15/299 | [0.01; 0.06] | 7 | 0.37 |
| TIA/Stroke | 18 | 0 | 3/589 | [0.00; 0.00] | 0 | 1.00 |
| Hospitalization  due to HF | 11 | 7 | 37/391 | [0.02; 0.13] | 50 | 0.03 |
| Bleeding | 19 | 10 | 54/593 | [0.05; 0.16] | 62 | <0.01 |
| Valve thrombosis | 10 | 5 | 11/182 | [0.01; 0.111 | 37 | 11 |
| AKI | 18 | 2 | 25/589 | [0.00; 0.05] | 49 | 0.01 |
| **Change Metrics** | **NO. of  Included articles** | **Odds Ratio or Mean Difference** | **P value** | **95% CI** | **I2** | **P value for heterogenicity** |
| NYHA class II or IV | 17 | 0.03 | < 0.01 | [0.01; 0.05] | 35 | 0.07 |
| TR grade ≥ 3 | 17 | 0.00 | < 0.01 | [0.00; 0.00] | 31 | 0.11 |
| 6MWT | 9 | 82.24 | <0.01 | [ 37.31; 127.18] | 90 | <0.01 |
| LVEF (%) | 9 | 0.96 | 0.47 | [-1.98; 3.90] | 65 | <0.01 |
| RV FAC (%) | 13 | -2.52 | 0.15 | [-6.09; 1.04] | 96 | < 0.01 |
| PASP (mm) | 6 | ﻿-8.69 | < 0.01 | [-11.54; -5.84] | 58 | 0.03 |
| TAPSE (mm) | 15 | -0.98 | 0.18 | [-2.48; 0.53] | 91 | < 0.01 |
| RV end-diastolic  base diameter | 9 | -6.33 | < 0.01 | [-8.92; -3.75] | 58 | 0.01 |
| RV end-diastolic mid diameter | 9 | -6.85 | < 0.01 | [-8.18; -5.52] | 5 | 0.4 |
| RV volume | 4 | -22.91 | < 0.01 | [-29.64; -16.171 | 0 | 0.64 |
| RA volume | 8 | -20.29 | < 0.01 | [-31.85; -8.73] | 29 | 0.20 |
| TV mean gradient | 8 | 0.20 | 0.78 | [-1.42; 1.81] | 94 | 0.01 |
| IVC diameter | 7 | -7.00 | < 0.01 | [-9.85; -4.14] | 88 | < 0.01 |
| Ascites | 4 | 0.11 | < 0.01 | [0.06; 0.24] | 0 | 0.79 |
| Edema | 7 | 0.09 | 0.02 | [0.01; 0.62] | 71 | <0.01 |

**Supplementary Table 5: Leave-One-Out Sensitivity Analysis of 30-Day Mortality Proportion following TTVR**

(Each row displays the recalculated mortality proportion and 95% confidence interval after exclusion of the corresponding study)

| **Removed Study** | **Total Events** | **Total N** | **Proportion** | **95% CI Lower** | **95% CI Upper** |
| --- | --- | --- | --- | --- | --- |
| None (All Included) | 21 | 487 | **0.0431** | 0.0284 | 0.065 |
| Kodali, 2023 | 17 | 311 | **0.0547** | 0.0344 | 0.0858 |
| Kodali, 2022 | 19 | 431 | **0.0441** | 0.0284 | 0.0678 |
| Fam, 2021 | 21 | 462 | **0.0455** | 0.0299 | 0.0685 |
| Hagemeyer, 2024 | 18 | 449 | **0.0401** | 0.0255 | 0.0625 |
| Hahn, 2020 | 17 | 457 | **0.0372** | 0.0234 | 0.0588 |
| Mao, 2022 | 20 | 472 | **0.0424** | 0.0276 | 0.0645 |
| Wang, 2024 | 20 | 467 | **0.0428** | 0.0279 | 0.0652 |
| Webb, 2022 | 21 | 460 | **0.0457** | 0.0301 | 0.0688 |
| Wei, 2022 | 20 | 457 | **0.0438** | 0.0285 | 0.0666 |
| Yu, 2022 | 20 | 470 | **0.0426** | 0.0277 | 0.0648 |
| Hahn, 2019 | 20 | 482 | **0.0415** | 0.027 | 0.0632 |
| Elgharably 2019 | 21 | 483 | **0.0435** | 0.0286 | 0.0656 |
| Mao, 2023 | 21 | 481 | **0.0437** | 0.0287 | 0.0658 |
| Sun, 2021 | 21 | 481 | **0.0437** | 0.0287 | 0.0658 |
| Fam, 2024 | 19 | 467 | **0.0407** | 0.0262 | 0.0627 |
| Lu, 2020 | 20 | 475 | **0.0421** | 0.0274 | 0.0641 |

**Supplementary Table 6:** Leave-One-Out Sensitivity Analysis of 1-year Mortality Proportions **following TTVR**

*(Each row shows the recalculated mortality proportion and 95% confidence interval after removing the indicated study)*

| **Removed Study** | **Total Events** | **Total N** | **Proportion** | **95% CI Lower** | **95% CI Upper** |
| --- | --- | --- | --- | --- | --- |
| None (All Included) | 25 | 268 | **0.0933** | 0.064 | 0.1341 |
| Kodali, 2023 | 9 | 92 | **0.0978** | 0.0523 | 0.1756 |
| Mao, 2022 | 24 | 253 | **0.0949** | 0.0646 | 0.1373 |
| Webb, 2022 | 23 | 241 | **0.0954** | 0.0644 | 0.1391 |
| Mao, 2023 | 25 | 262 | **0.0954** | 0.0655 | 0.1371 |
| Sun, 2021 | 24 | 262 | **0.0916** | 0.0623 | 0.1327 |
| Stoltz, 2023 | 20 | 230 | **0.087** | 0.057 | 0.1305 |

**Supplementary Table 7:** Leave-One-Out Sensitivity Analysis of Severe Tricuspid Regurgitation (TR) or TR > Grade II/III Before and After TTVR

(Odds ratios calculated after excluding each study sequentially)

| **Study Name** | **Sever TR (After)** | **Total (After)** | **Sever TR (Before)** | **Total (Before)** | **Odds Ratio (OR)** | **95% CI** | **P-value** |
| --- | --- | --- | --- | --- | --- | --- | --- |
| Kodali et al. 2023 | 0 | 176 | 155 | 176 | **0.00** | [0.00, 0.00] | **<0.001** |
| Kodali et al. 2022 | 0 | 56 | 51 | 56 | **0.00** | [0.00, 0.00] | **<0.001** |
| Fam et al. 2021 | 1 | 25 | 25 | 25 | **0.00** | [0.00, 0.00] | **<0.001** |
| Hagemeyer et al. 2024 | 0 | 38 | 38 | 38 | **0.00** | [0.00, 0.00] | **<0.001** |
| Hahn et al. 2020 | 4 | 30 | 28 | 30 | **0.00** | [0.00, 0.00] | **<0.001** |
| Lu et al. 2021 | 0 | 46 | 46 | 46 | **0.00** | [0.00, 0.00] | **<0.001** |
| Mao et al. 2022 | 0 | 15 | 15 | 15 | **0.00** | [0.00, 0.00] | **<0.001** |
| Ning et al. 2023 | 0 | 22 | 22 | 22 | **0.00** | [0.00, 0.00] | **<0.001** |
| Wang et al. 2024 | 0 | 20 | 20 | 20 | **0.00** | [0.00, 0.00] | **<0.001** |
| Webb et al. 2022 | 1 | 27 | 27 | 27 | **0.00** | [0.00, 0.00] | **<0.001** |
| Yu et al. 2022 | 1 | 17 | 17 | 17 | **0.00** | [0.00, 0.00] | **<0.001** |
| Elgharably et al. 2019 | 1 | 4 | 4 | 4 | **0.00** | [0.00, 0.00] | **<0.001** |
| Mao et al. 2023 | 0 | 6 | 6 | 6 | **0.00** | [0.00, 0.00] | **<0.001** |
| Sun et al. 2021 | 0 | 6 | 6 | 6 | **0.00** | [0.00, 0.00] | **<0.001** |
| Fam et al. 2024 | 0 | 20 | 20 | 20 | **0.00** | [0.00, 0.00] | **<0.001** |
| Lu et al. 2020 | 0 | 12 | 12 | 12 | **0.00** | [0.00, 0.00] | **<0.001** |
| Weckbach et al. 2023 | 0 | 25 | 25 | 25 | **0.00** | [0.00, 0.00] | **<0.001** |

**Supplementary Table 8:** Leave-One-Out Sensitivity Analysis of Pre- and Post-Procedure Pulmonary Artery Systolic Pressure (PASP)

(Mean differences (MD) were calculated after removing each study individually)

| **Study** | **Pre-Mean** | **Pre-SD** | **Pre-N** | **Post-Mean** | **Post-SD** | **Post-N** | **Mean Difference (MD)** | **95% CI** | **P-value** |
| --- | --- | --- | --- | --- | --- | --- | --- | --- | --- |
| Kodali, 2023 | 32.50 | 11.00 | 176.00 | 39.30 | 12.80 | 176.00 | **-9.39** | [-12.20, -6.58] | **<0.001** |
| Kodali, 2022 | 32.20 | 10.20 | 56.00 | 40.10 | 10.50 | 56.00 | **-8.81** | [-11.81, -5.80] | **<0.001** |
| Dershowitz, 2023 | 34.46 | 13.04 | 25.00 | 44.06 | 13.59 | 25.00 | **-8.56** | [-11.30, -5.83] | **<0.001** |
| Hahn, 2020 | 37.00 | 9.34 | 30.00 | 41.00 | 19.46 | 30.00 | **-9.1** | [-11.65, -6.55] | **<0.001** |
| Wang, 2024 | 33.60 | 4.70 | 20.00 | 45.80 | 2.60 | 20.00 | **-7.26** | [-9.11, -5.41] | **<0.001** |
| Webb, 2022 | 34.90 | 10.00 | 27.00 | 43.60 | 11.80 | 27.00 | **-8.64** | [-11.47, -5.80] | **<0.001** |

**Supplementary Table 9:** Leave-One-Out Sensitivity Analysis of Pre- and Post-Procedure Right Ventricular End-Diastolic Base Diameter

*(Mean differences (MD) were calculated after removing each study individually)*

| **Study** | **Pre-Mean** | **Pre-SD** | **Pre-N** | **Post-Mean** | **Post-SD** | **Post-N** | **Mean Difference (MD)** | **95% CI** | **P-value** |
| --- | --- | --- | --- | --- | --- | --- | --- | --- | --- |
| Fam, 2021 | 46.4 | 6.3 | 25 | 50.8 | 7.2 | 25 | **-6.3** | [-7.82, -4.79] | **<0.001** |
| Hagemeyer, 2024 | 50.66 | 10.01 | 38 | 53.16 | 6.28 | 38 | **-6.52** | [-8.09, -4.95] | **<0.001** |
| Lu, 2021 | 48.23 | 8.57 | 46 | 54.13 | 9.64 | 46 | **-6.61** | [-8.12, -5.10] | **<0.001** |
| Mao, 2022 | 48.66 | 13.16 | 15 | 55.7 | 20.2 | 15 | **-5.76** | [-7.20, -4.31] | **<0.001** |
| Wang, 2024 | 43.5 | 1.9 | 20 | 56.2 | 8.7 | 20 | **-6.06** | [-7.57, -4.55] | **<0.001** |
| Webb, 2022 | 44.7 | 7.7 | 27 | 50.7 | 7.3 | 27 | **-6.02** | [-7.44, -4.61] | **<0.001** |
| Hahn, 2019 | 43.75 | 4.32 | 5 | 54.4 | 5.08 | 5 | **-6.04** | [-7.49, -4.60] | **<0.001** |
| Mao, 2023 | 51.25 | 4.12 | 6 | 57.2 | 6.24 | 6 | **-5.05** | [-6.55, -3.54] | **<0.001** |
| Fam, 2024 | 50.1 | 5.2 | 20 | 54.2 | 4.9 | 20 | **-6.04** | [-7.54, -4.54] | **<0.001** |

**Supplementary Table 10:** Leave-One-Out Sensitivity Analysis of Pre- and Post-Procedure Right Ventricular End-Diastolic Mid-Diameter

*(Mean differences (MD) were calculated after removing each study individually)*

| **Study** | **Pre-Mean** | **Pre-SD** | **Pre-N** | **Post-Mean** | **Post-SD** | **Post-N** | **Mean Difference (MD)** | **95% CI** | **P-value** |
| --- | --- | --- | --- | --- | --- | --- | --- | --- | --- |
| Kodali, 2023 | 35 | 7.4 | 176 | 41 | 8.8 | 176 | **-6.96** | [-8.57, -5.34] | **<0.001** |
| Kodali, 2022 | 23 | 7.1 | 56 | 33 | 7.2 | 56 | **-6.32** | [-7.46, -5.17] | **<0.001** |
| Dershowitz, 2023 | 34 | 6 | 25 | 38 | 7.2 | 25 | **-7.14** | [-8.40, -5.88] | **<0.001** |
| Hagemeyer, 2024 | 38 | 6.2 | 38 | 42 | 11 | 38 | **-7.05** | [-8.37, -5.73] | **<0.001** |
| Mao, 2022 | 36 | 10 | 15 | 43 | 12 | 15 | **-6.86** | [-8.16, -5.56] | **<0.001** |
| Wang, 2024 | 37 | 5.2 | 20 | 44 | 5.4 | 20 | **-6.72** | [-8.16, -5.27] | **<0.001** |
| Webb, 2022 | 33 | 6.9 | 27 | 40 | 7.4 | 27 | **-6.85** | [-8.27, -5.43] | **<0.001** |
| Hahn, 2019 | 33 | 2.5 | 5 | 40 | 3.9 | 5 | **-6.78** | [-8.18, -5.38] | **<0.001** |
| Mao, 2023 | 38 | 4.1 | 6 | 45 | 4.7 | 6 | **-6.84** | [-8.20, -5.48] | **<0.001** |


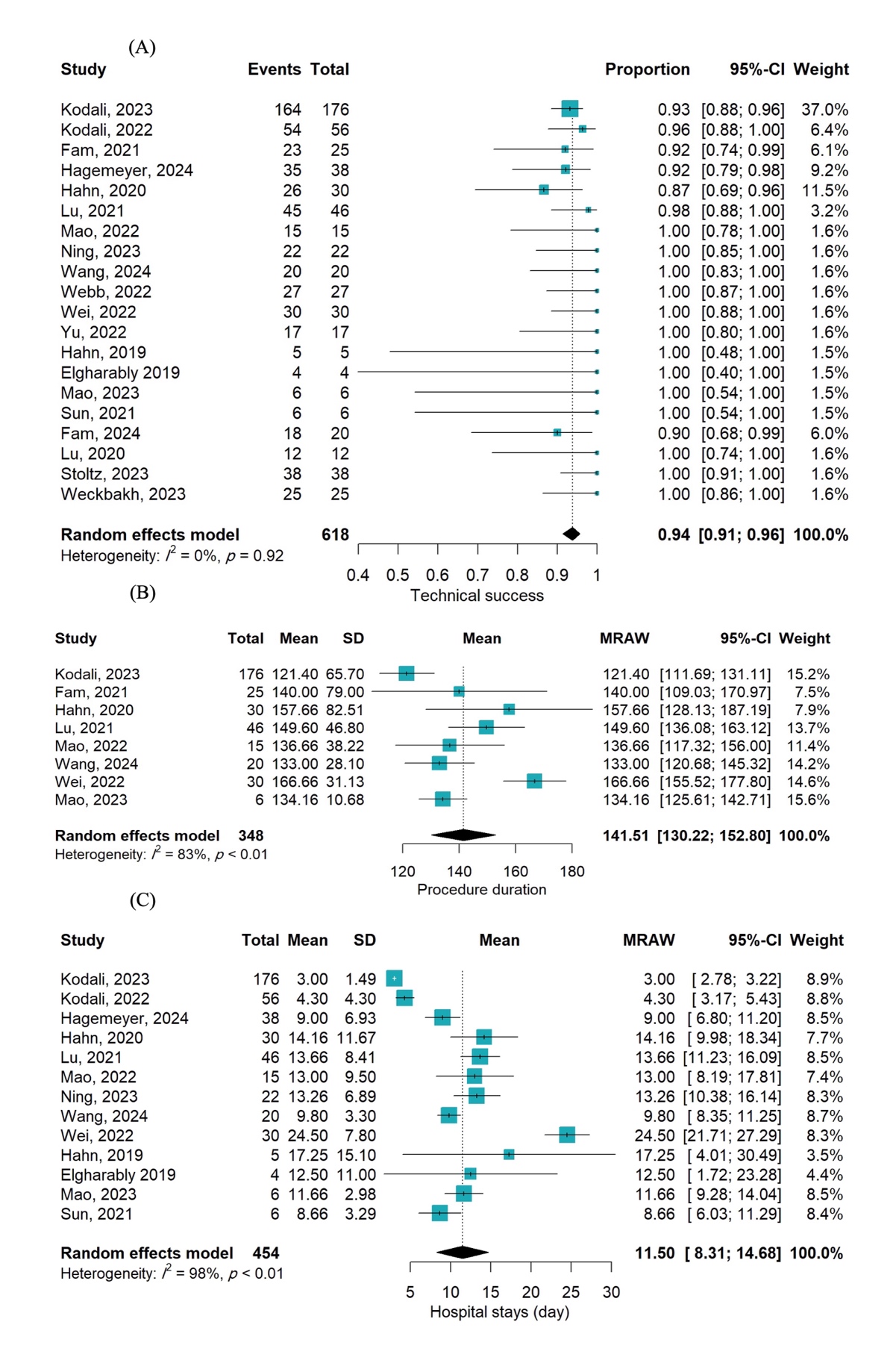


**Supplementary Figure 1:** (A) Forest plot illustrating the proportion of technical success following TTVR. (B) Forest plot illustrating the mean procedure duration time following TTVR. (C) Forest plot illustrating the mean hospital stay following TTVR.


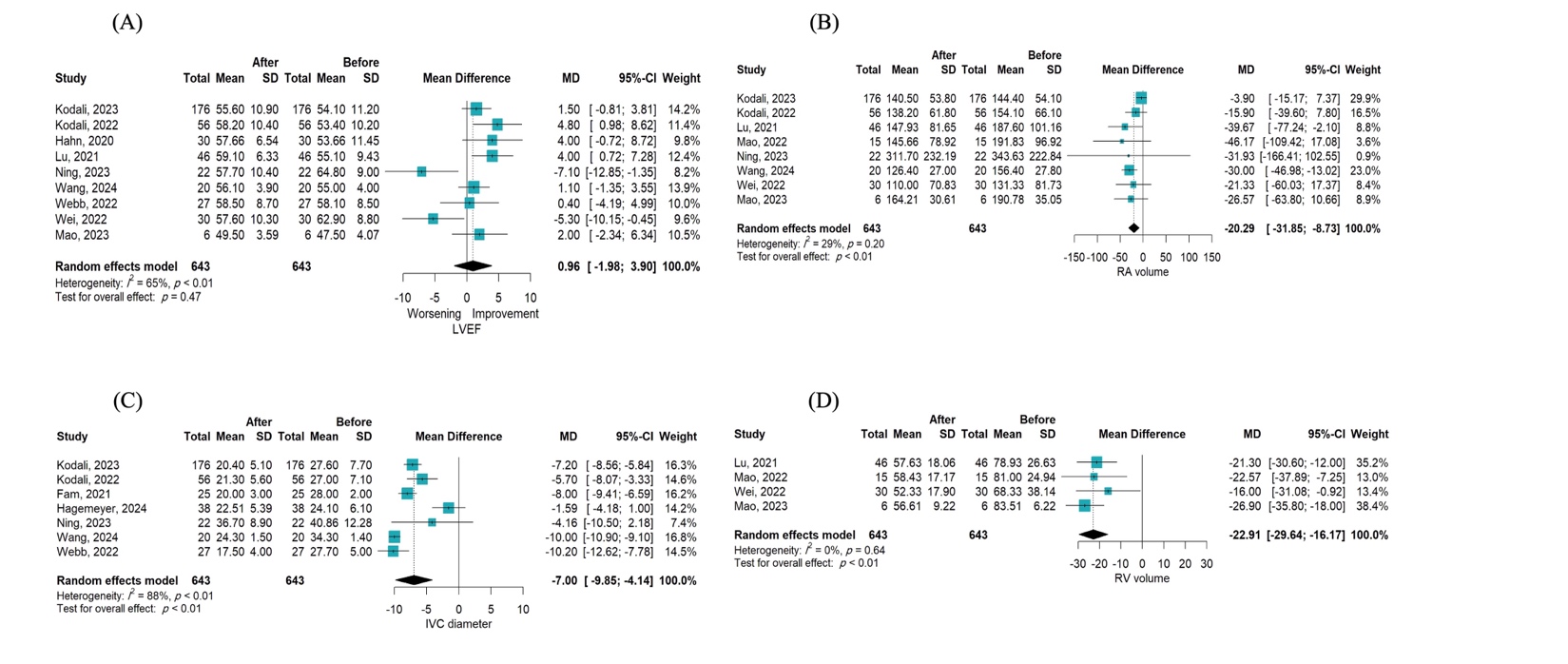


**Supplementary Figure 2:** (A) Forest plot illustrating the mean difference in LVEF at baseline versus last follow-up. (B) Forest plot illustrating the mean difference in RA volume at baseline versus last follow-up. (C) Forest plot illustrating the mean difference in IVC diameter at baseline versus last follow-up. (D) Forest plot illustrating the mean difference in RV volume at baseline versus last follow-up. LVEF: left ventricular ejection fraction; RA: right atrium; IVC: inferior vena cava; RV: right ventricle.


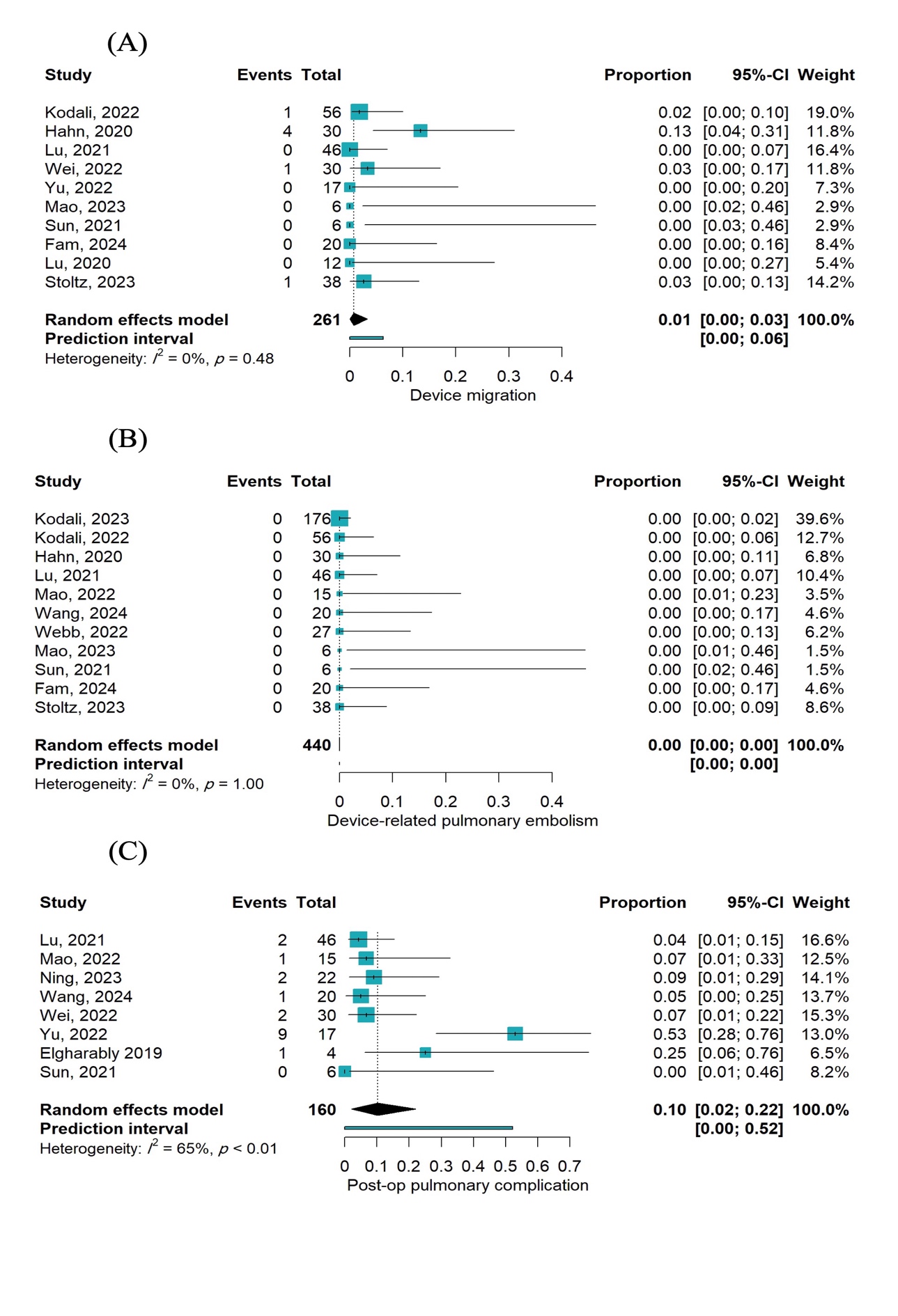


**Supplementary Figure 3:** (A) Forest plot illustrating the proportion of device migration following TTVR. (B) Forest plot illustrating the proportion of device-related pulmonary embolism following TTVR. (C) Forest plot illustrating the proportion of post-operation pulmonary complications following TTVR.


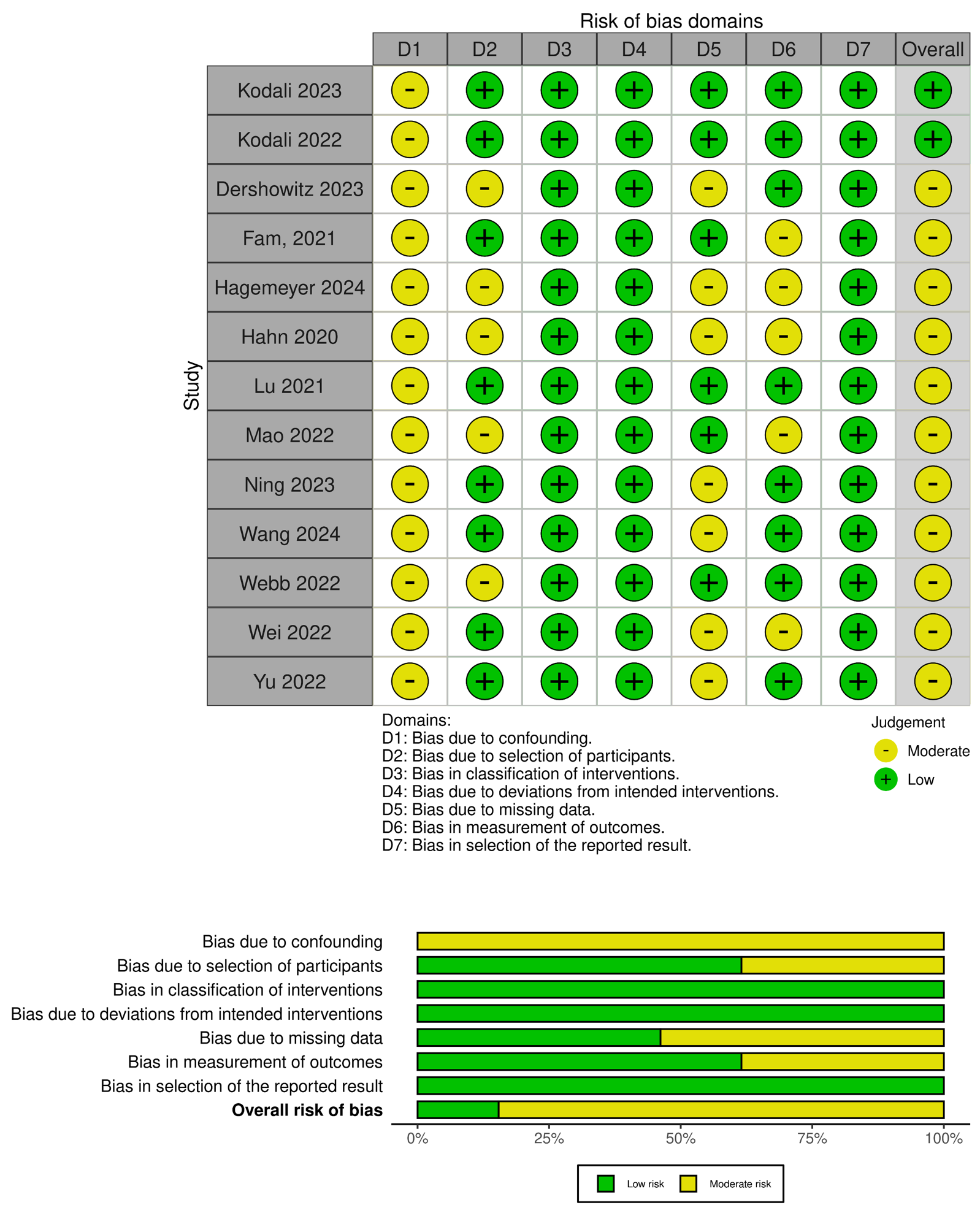


**Supplementary Figure 4:** Risk of Bias Assessment Using ROBINS-I Tool for Included Observational Studies.


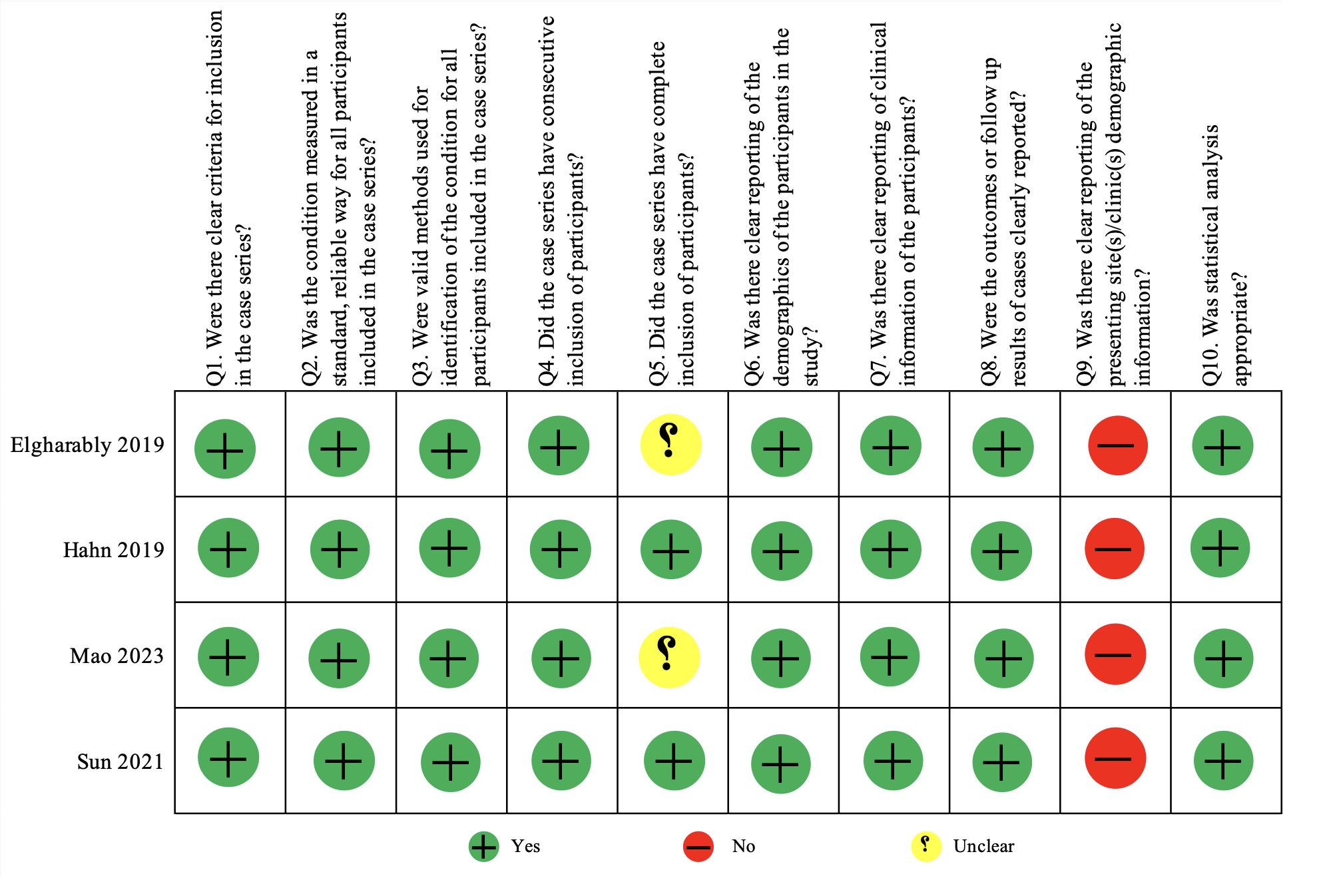


**Supplementary Figure 5:** Risk of Bias Assessment Using JBI Checklist for Included Case Series Studies


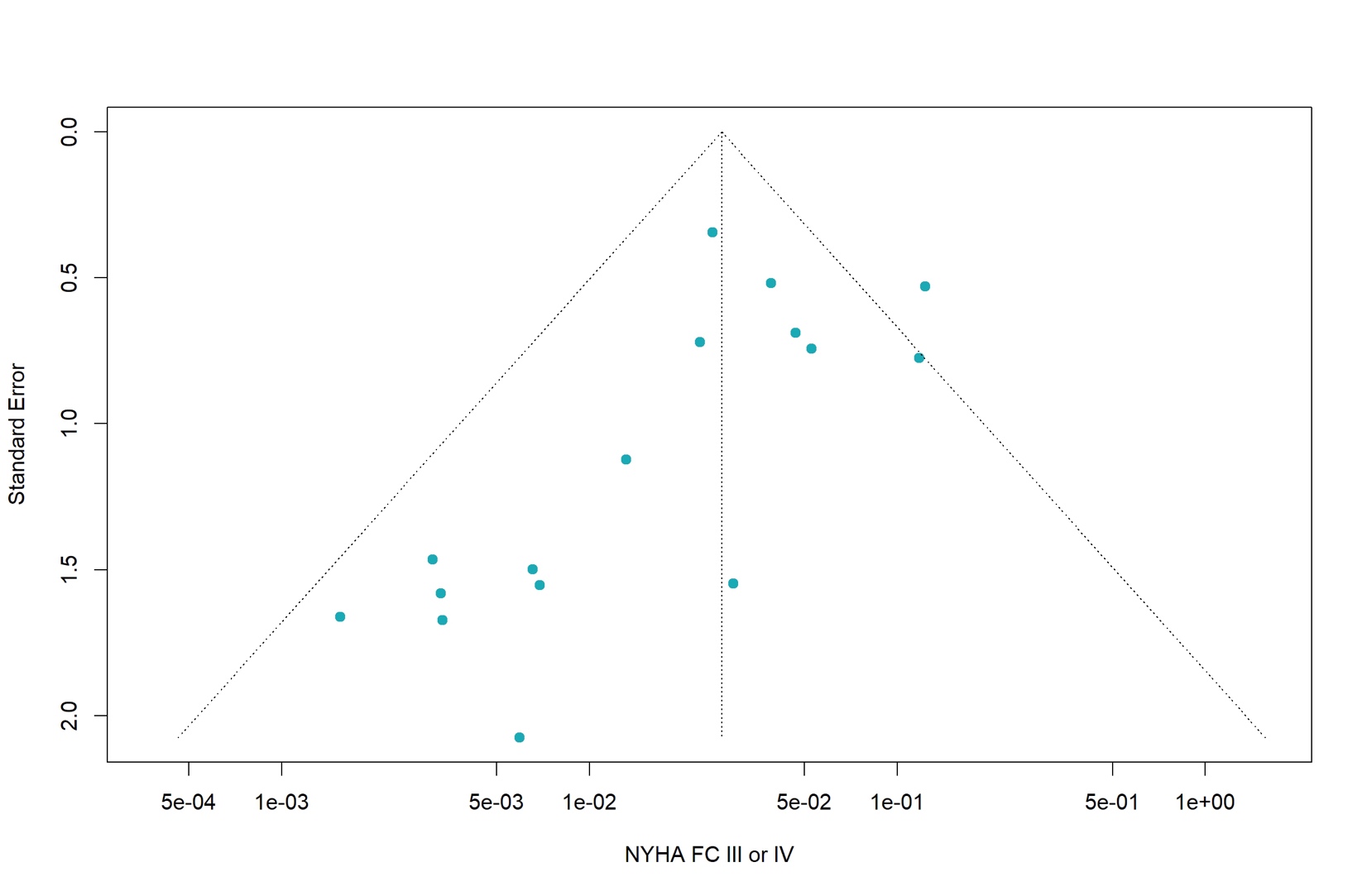


**Supplementary Figure 6:** Funnel plot assessing the publication bias of the NYHA FC III or IV. The blue dots indicate the individual studies included in the meta-analysis. 6MWD: 6-minutes walking distance; NYHA: New York Heart Association; FC: functional class


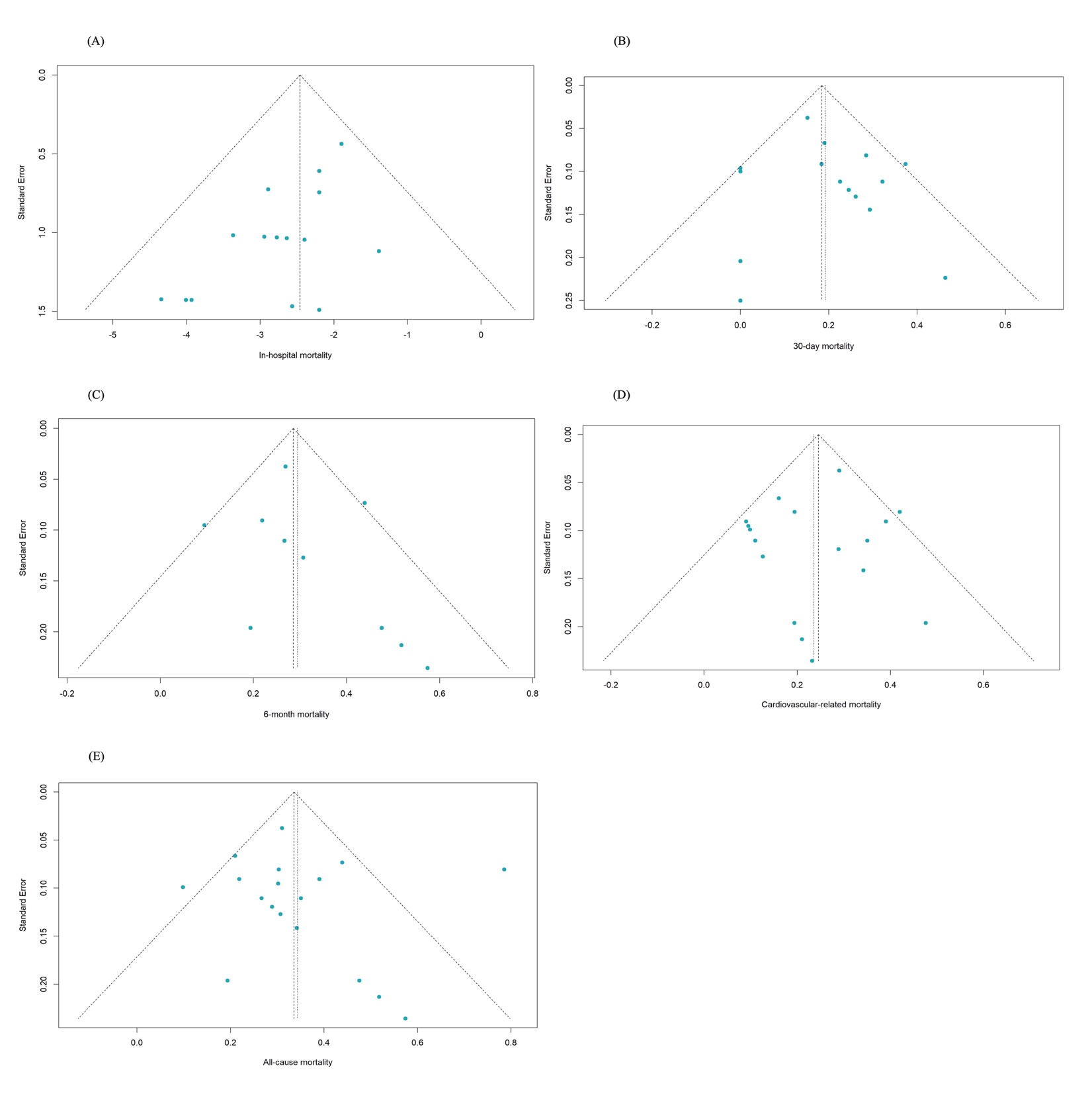


**Supplementary Figure 7:** Funnel plot assessing publication bias for the outcomes of interest. The blue dots represent individual studies included in the meta-analysis. (A) In-hospital mortality; (B) 30-day mortality; (C) 6-month mortality; (D) Cardiovascular-related mortality; (E) All-cause mortality.


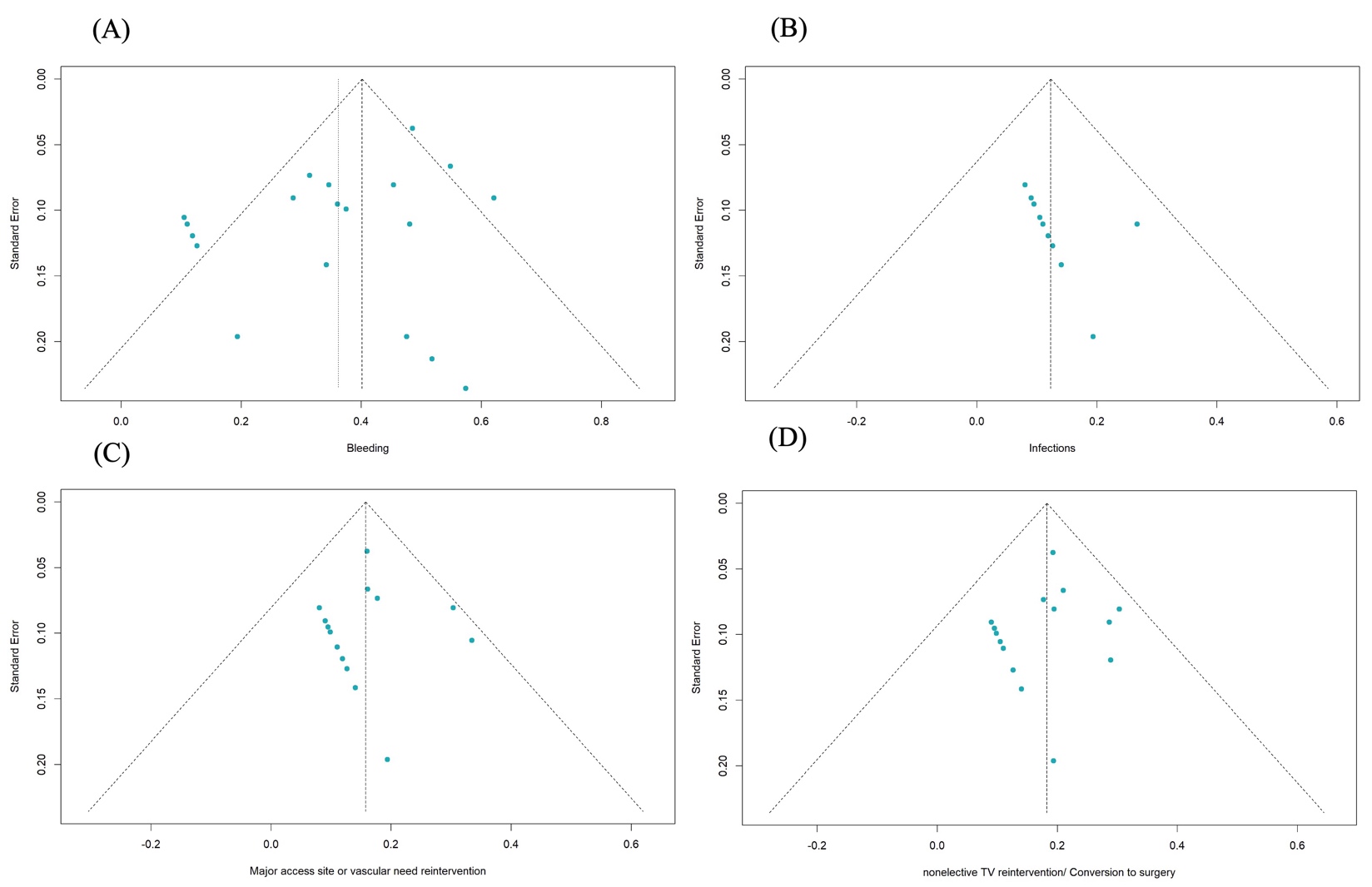


**Supplementary Figure 8:** Funnel plot assessing publication bias for the outcomes of interest. The blue dots represent individual studies included in the meta-analysis. (A) Bleeding; (B) Infection; (C) major access site or vascular reintervention; (D) nonelective TV reintervention/conversion to surgery.


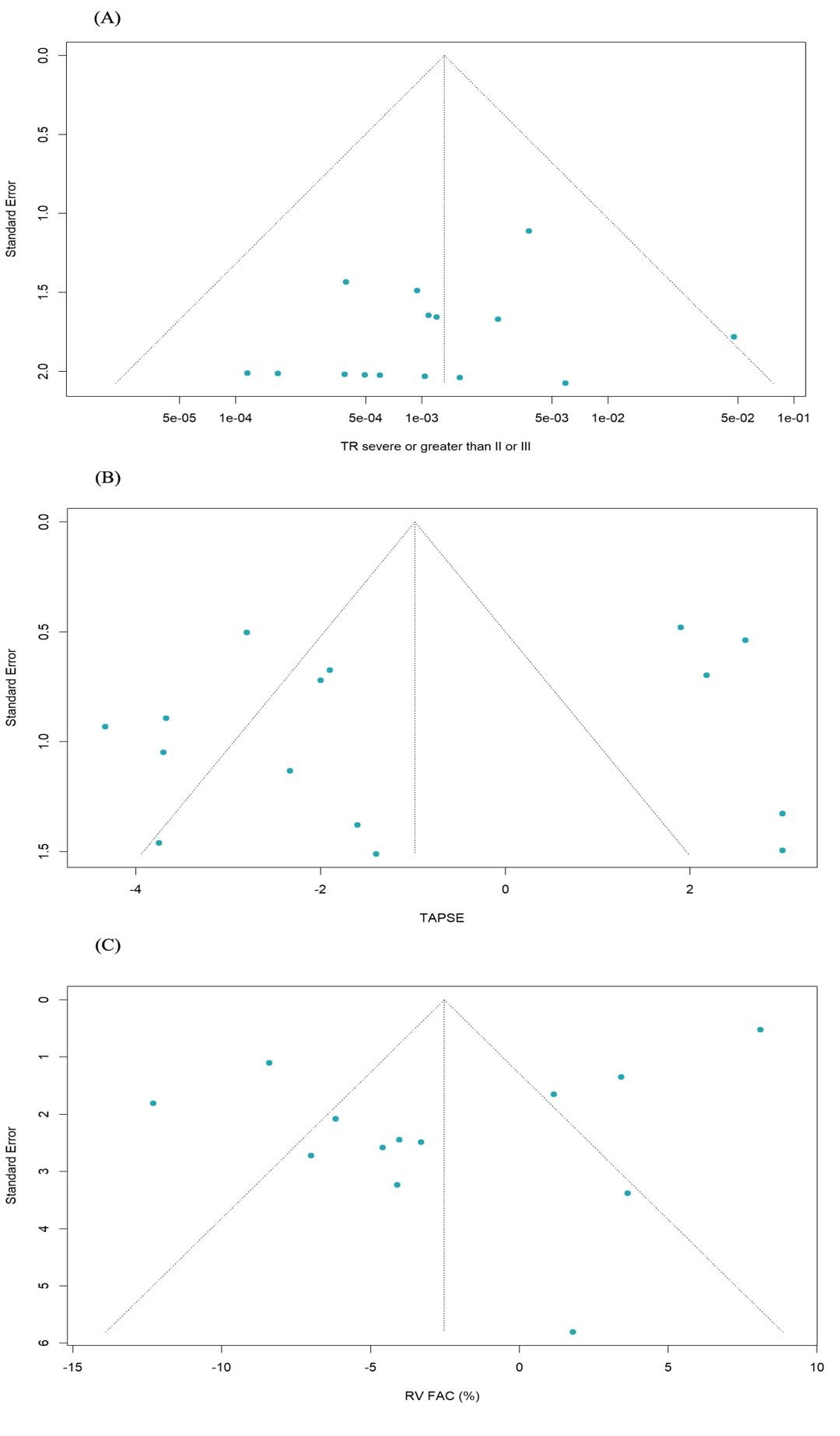


**Supplementary Figure 9:** Funnel plot assessing publication bias for the outcomes of interest. The blue dots represent individual studies included in the meta-analysis. (A) TR severe or greater than grade II or III; (B) TAPSE; (C) RV FAC (%). TR: tricuspid regurgitation; TAPSE: tricuspid annular plane systolic excursion; RV: right ventricle; FAC: fractional area change


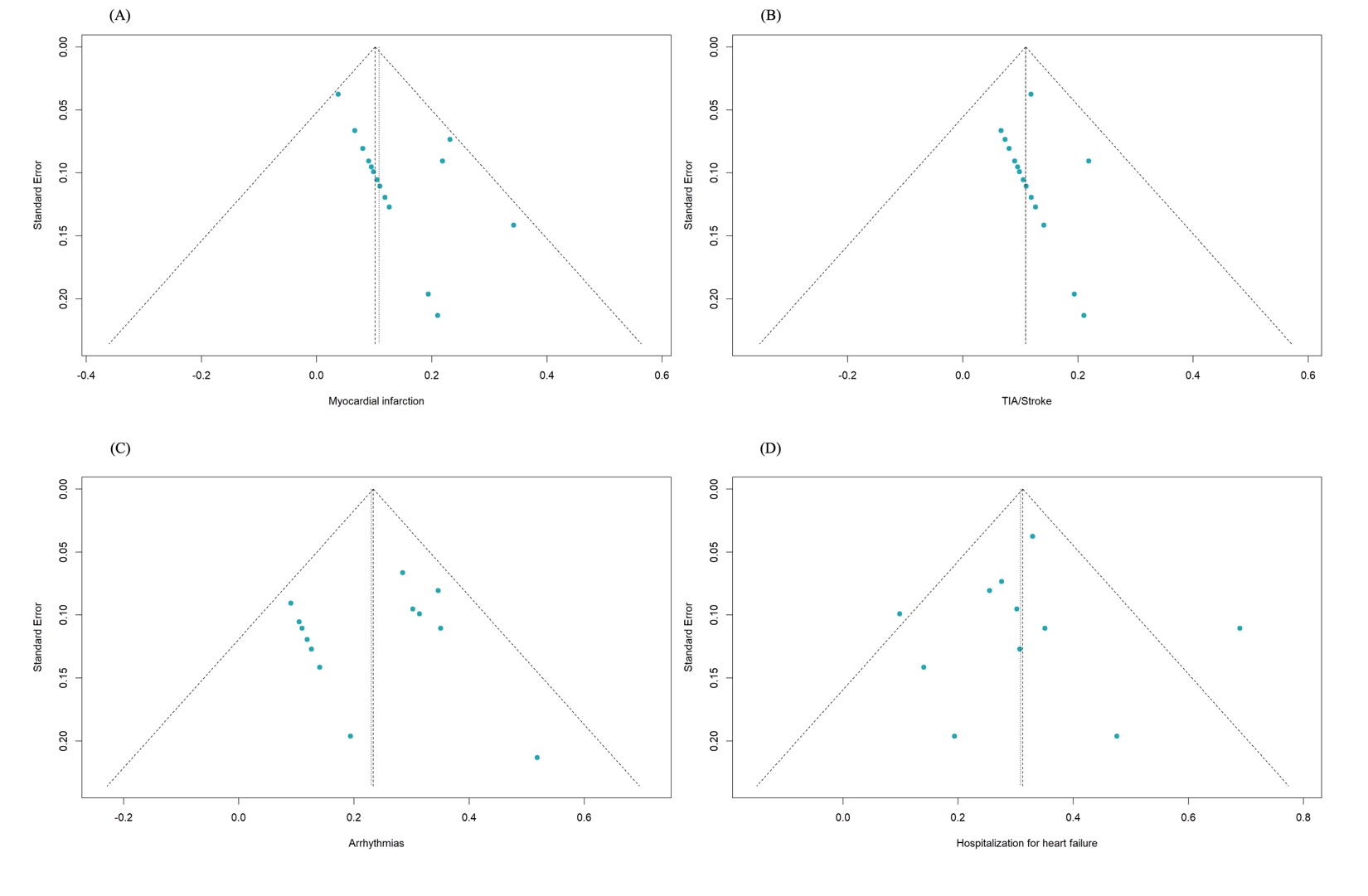


**Supplementary Figure 10:** Funnel plot assessing publication bias for the outcomes of interest. The blue dots represent individual studies included in the meta-analysis. (A) myocardial infarction; (B) TIA/stroke; (C) arrhythmias; (D) hospitalization due to heart failure. TIA: transient ischemic accident.


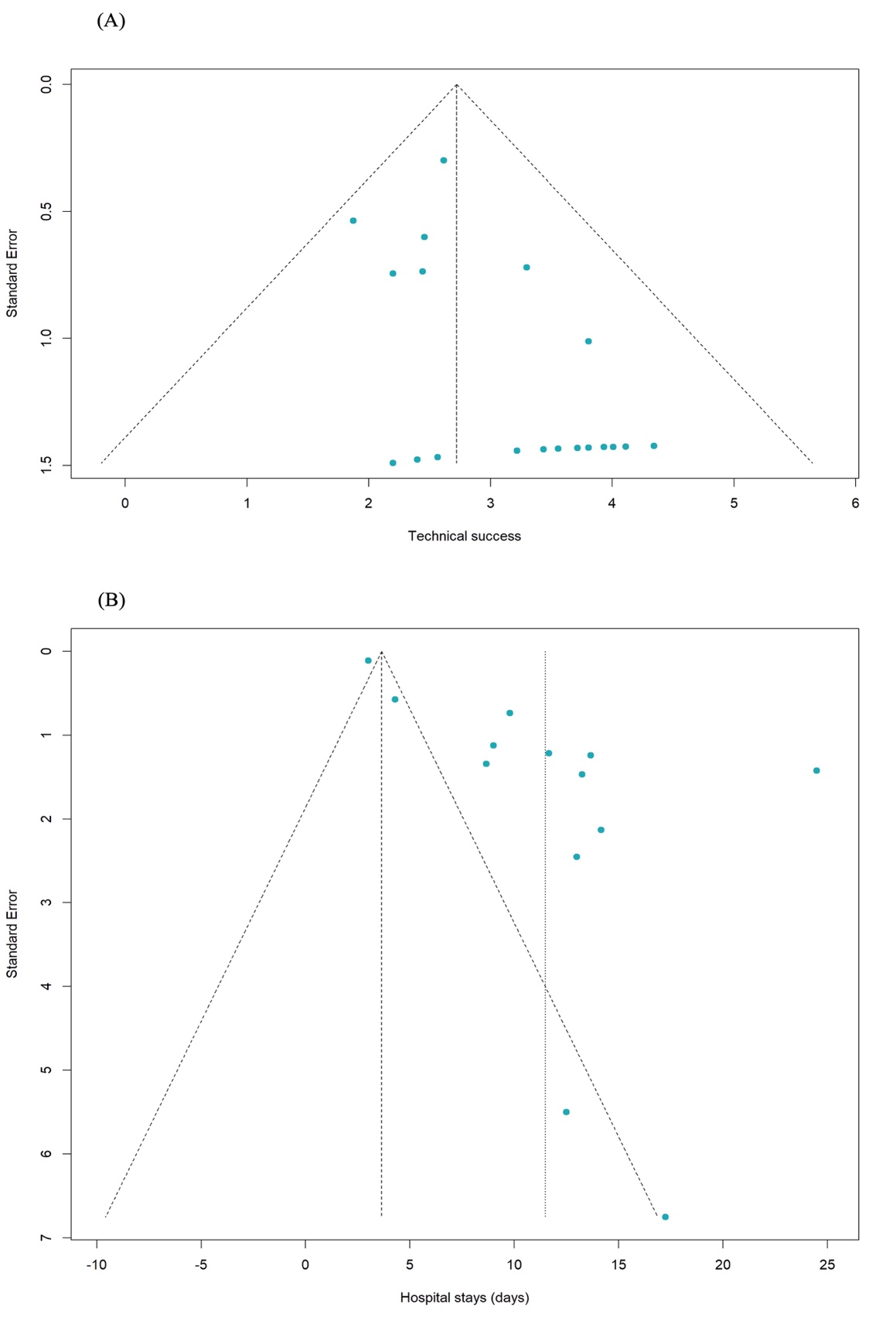


**Supplementary Figure 11:** Funnel plot assessing publication bias for the outcomes of interest. The blue dots represent individual studies included in the meta-analysis. (A) technical success; (B) hospital stays.
